# Structural basis of the regulation of the normal and oncogenic methylation of nucleosomal histone H3 Lys36 by NSD2

**DOI:** 10.1038/s41467-021-26913-5

**Published:** 2021-11-15

**Authors:** Ko Sato, Amarjeet Kumar, Keisuke Hamada, Chikako Okada, Asako Oguni, Ayumi Machiyama, Shun Sakuraba, Tomohiro Nishizawa, Osamu Nureki, Hidetoshi Kono, Kazuhiro Ogata, Toru Sengoku

**Affiliations:** 1 Department of Biochemistry, Yokohama City University Graduate School of Medicine, 3-9 Fukuura, Kanazawa-ku, Yokohama, Kanagawa 236-0004 Japan; 2 Institute for Quantum Life Science, National Institutes for Quantum Science and Technology, 8-1-7 Umemidai, Kizugawa, Kyoto 619-0215 Japan; 3 Department of Biological Sciences, Graduate School of Science, The University of Tokyo, 7-3-1 Hongo, Bunkyo-ku, Tokyo 113-0033 Japan; 4 Present Address: Graduate School of Medical Life Science, Yokohama City University, 1-7-29 Suehiro-cho, Tsurumi-ku, Yokohama, Kanagawa 230-0045 Japan

**Keywords:** Chemical modification, Enzyme mechanisms, Cancer, Epigenetics, Cryoelectron microscopy

## Abstract

Dimethylated histone H3 Lys36 (H3K36me2) regulates gene expression, and aberrant H3K36me2 upregulation, resulting from either the overexpression or point mutation of the dimethyltransferase NSD2, is found in various cancers. Here we report the cryo-electron microscopy structure of NSD2 bound to the nucleosome. Nucleosomal DNA is partially unwrapped, facilitating NSD2 access to H3K36. NSD2 interacts with DNA and H2A along with H3. The NSD2 autoinhibitory loop changes its conformation upon nucleosome binding to accommodate H3 in its substrate-binding cleft. Kinetic analysis revealed that two oncogenic mutations, E1099K and T1150A, increase NSD2 catalytic turnover. Molecular dynamics simulations suggested that in both mutants, the autoinhibitory loop adopts an open state that can accommodate H3 more often than the wild-type. We propose that E1099K and T1150A destabilize the interactions that keep the autoinhibitory loop closed, thereby enhancing catalytic turnover. Our analyses guide the development of specific inhibitors of NSD2.

## Abstract (web summary)

An upregulation of NSD2, a histone H3 lysine 36 (H3K36) methyltransferase is linked to multiple myeloma and other types of cancer. Here, the authors provide insights into the regulatory mechanism of NSD2 by determining the 2.8 Å cryo-EM structure of the NSD2 bound nucleosome complex, and based on MD simulations they discuss how two oncogenic mutations enhance NSD2 activity.

## Introduction

Histones are subjected to a variety of posttranslational modifications that regulate diverse aspects of genome functions^1^. The dysregulation of histone modifications is often linked to diseases such as developmental defects and cancers^2^. NSD2 (also known as WHSC1/MMSET) is a member of the NSD family that catalyzes the mono- and dimethylation of histone H3 K36^3^. Dimethylated H3 K36 (H3K36me2) antagonizes the activity of polycomb repressive complex 2 (PRC2) in catalyzing H3K27 trimethylation, a hallmark of repressive chromatin^4^. Therefore, H3K36me2 maintains gene expression by protecting genomic regions from the spreading of repressive chromatin domains. Moreover, H3K36me2 serves as a binding site for DNMT3A, a de novo DNA methyltransferase, thereby controlling the DNA methylation pattern mainly in intergenic regions^5^.

Several lines of evidence have demonstrated the critical importance of the strict regulation of cellular H3K36me2 levels. First, haploinsufficiency of NSD2 or NSD1 is involved in Wolf–Hirschhorn syndrome or Sotos syndrome, respectively^6,7^. Second, aberrant upregulation of cellular H3K36me2 levels, induced by the overexpression or point mutation of NSD2, has been found in various cancers. Approximately 15–20% of patients with multiple myeloma carry a t(4;14) translocation, which induces NSD2 overexpression from an immunoglobulin heavy chain locus *(IGH)-NSD2* hybrid, along with a global increase and redistribution of H3K36me2 in the affected cells^8^. An increased H3K36me2 level reprograms cells by reversing the repressive function of PRC2 in myeloma^9^. A recurrent point mutation in the catalytic SET domain, NSD2 p.E1099K, has been found in patients with acute lymphoblastic leukemia^10^ and other types of cancers^11^ and is known to aberrantly activate H3K36 methyltransferase activity^10,11^. Another recurrent mutation in the SET domain, NSD2 p.T1150A, has been found in mantle cell lymphoma along with the p.E1099K mutation^12^ and was recently shown to be catalytically hyperactivated^13^.

Biochemical studies have revealed that nucleosome structures regulate the methylation activity of NSD proteins. NSD2 exhibits weak and nonspecific lysine methylation activity on histone octamer substrates, whereas it strongly and specifically methylates H3K36 when nucleosomal substrates are used^14^. The linker histone H1, on the other hand, inhibits the activity of NSD2 on nucleosomal substrates^15,16^. Crystal structures of the SET domains of NSD1^17^, NSD2^18^, and NSD3 (PDB 5UPD) have shown the substrate-binding cleft to be occupied by the autoinhibitory loop (residues 1180–1188 of NSD2), preventing H3K36 binding. The structure of Set2, an H3K36 trimethyltransferase, bound to the nucleosome has been reported^19^, revealing that Set2 captures nucleosomes with partially unwrapped DNA. It has been unclear whether NSD2 captures partially unwrapped nucleosomes similarly to Set2 and how the oncogenic mutations alter its catalytic activity.

To understand how NSD2 engages with nucleosomal H3K36 and how the oncogenic mutations affect its catalytic activity, we report the structure of the NSD2-nucleosome complex. NSD2 binds to the nucleosome with partially unwrapped DNA and interacts with DNA and H2A, in addition to H3. Upon nucleosome binding, the autoinhibitory loop of NSD2 changes its conformation to accommodate the H3 tail in the catalytic cleft. We also show that the oncogenic E1099K and T1150A mutants exhibit increased catalytic turnover but not an increased nucleosome affinity. Molecular dynamics simulations suggest that E1099K and T1150A increase the tendency of the autoinhibitory loop to adopt an open state. Our analyses provide insights into the regulatory mechanisms of NSD proteins under normal and oncogenic conditions and pave the way for the development of inhibitors of these proteins for the treatment of cancers.

## Results

### Overall structure of the NSD2-nucleosome complex

We solved a 2.8-Å resolution cryo-electron microscopy (cryo-EM) structure of the complex formed by the catalytic fragment of human NSD2 bearing an E1099K mutation (residues 973–1226, containing the AWS domain, SET domain, and C-terminal basic extension) and a nucleosome in the presence of sinefungin, an analog of *S*-adenosyl methionine (SAM) (Supplementary Figs. 1, 2, Supplementary Table 1). To facilitate complex formation, we created an NSD2-H4 fusion protein connected by a 32-residue linker and coexpressed it with H3. We then assembled a nucleosome using the coexpressed proteins, along with H2A, H2B, and a 185-bp DNA fragment possessing the 601 nucleosome positioning sequence at its center and 20-bp linker DNA sequences at both ends. We additionally introduced H3K36M substitution that is found in most patients with chondroblastoma^20^, is known to inhibit several H3K36 methyltransferases^21^, and has been used in structural studies of Set2^19^ and SETD2^22^.

**Fig. 1:**
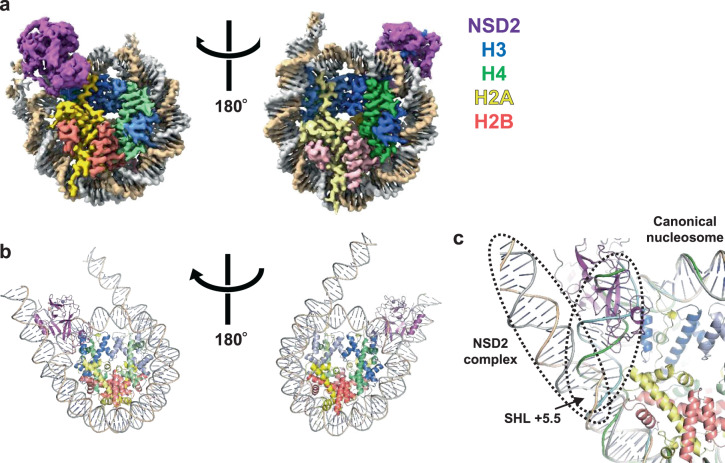
Overall structure. Unless stated otherwise, proteins are colored as follows: NSD2, magenta; H3, blue; H4, green; H2A, pink; and H2B, yellow. **a** Density map and **b** ribbon model, two views related by 180° rotation. **c** Superposition of the current NSD2-nucleosome structure with that of a canonical nucleosome (PDB 1KX5). Duplex DNA in the NSD2 complex stretches straight up to approximately SHL +5.5, resulting in its partial unwrapping.

Fig. 1 shows the overall structure of the NSD2-nucleosome complex. Although a single nucleosome should contain two copies of NSD2 (due to the NSD2-H4 fusion used), we observed only one NSD2 molecule engaging with one of the two H3K36M residues; the other molecule may exhibit a random orientation with respect to the nucleosome position. Judging from the density pattern of purine-pyrimidine base pairs at several positions (Supplementary Fig. 3), NSD2 preferentially binds to the DNA end of the 601 sequence known to be unwrapped more easily^23^. This suggests that nucleosomes with flexible DNA are more susceptible to H3K36 methylation by NSD2, indicating a possible mechanism by which DNA sequence regulates NSD2’s activity. Residues 986–1203 of NSD2 and 31–134 of the engaged H3 are visible in cryo-EM density and have been included in the model coordinates. The basic C-terminal extension (residues 1209–1226) is required for efficient nucleosome H3K36 methylation by NSD proteins^24^. Although no clear density was observed, the extension is expected to be located near nucleosomal DNA and to form ionic interactions with the DNA phosphates. Upon nucleosome binding, NSD2 was seen to markedly change its conformation at the autoinhibitory loop to allow the accommodation of the H3 tail (see “Conformational change of the autoinhibitory loop”).

**Fig. 2:**
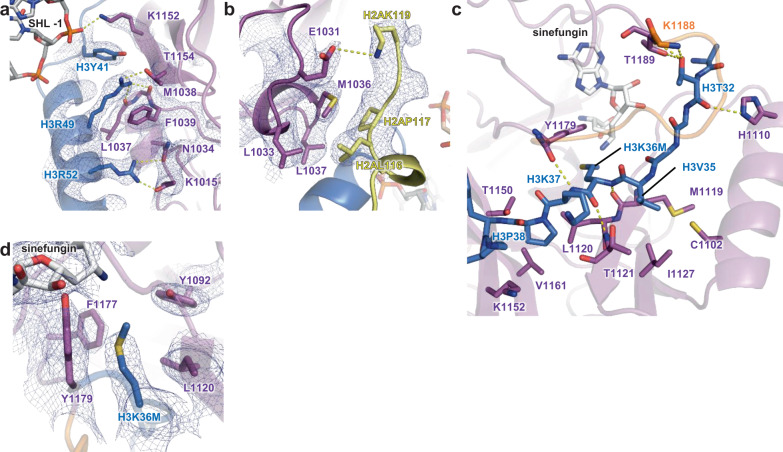
Interactions of NSD2 with histones H3 and H2A and DNA. The cryo-EM density is shown as a blue mesh in **a** (contoured at 7σ), **b** (at 3σ), and **d** (at 5σ). **a** Interactions with the first α-helix of H3 and DNA. **b** Interactions with H2A. **c** Interactions with the H3 tail region. **d** The H3K36-binding cavity.

In the complex structure, the nucleosomal DNA is partially unwrapped near the entry site (Fig. 1a, c), as observed in the Set2-nucleosome complex^19^. In the canonical nucleosome, H3K36 is located between the two gyres of DNA in a relatively crowded environment. Thus, NSD2 needs to remove one gyre to gain access to H3K36 and accommodate its side chain in the catalytic pocket. Nucleosomal DNA, which usually exhibits a curved structure wrapping around the histone octamer, stretches around superhelix location (SHL) +5.5 and extrudes away from the histone octamer. This extrusion allows NSD2 to establish interactions with the first α-helix of H3. Moreover, NSD2 interacts with the N-terminal tail of H3, the C-terminal portion of H2A, and DNA at two locations, SHL-1 and the external linker DNA position (Fig. 2a and Supplementary Fig. 4a, b). The interaction modes are similar to those observed in the complex between yeast Set2, an H3K36 trimethyltransferase, and the nucleosome (Supplementary Fig. 4c)^19^. However, the linker DNA exhibits different conformations in the two complexes (Supplementary Fig. 4d), and three lysine residues of NSD2 (K992, K995, and K998), conserved across NSD proteins but not in Set2, face the linker DNA (Supplementary Fig. 4b, 5).

### Interactions of NSD2 with histones and DNA

Fig. 2 shows the interactions between NSD2 and histones in detail. Two arginine residues in the first α-helix of H3 (H3R49 and H3R52) make extensive contact with NSD2 (Fig. 2a). In the canonical nucleosome, these arginine residues are located close to the phosphate groups of DNA. The interactions between NSD2 and the two arginine residues may compensate for the loss of their interactions with DNA. In addition, H3Y41 stacks against K1152, which further forms a salt bridge with the phosphate group of nucleosomal DNA at SHL -1.

The interactions between NSD2 and H2A are mainly hydrophobic, and there is a salt bridge between E1031 and H2AK119 (Fig. 2b). H2AK119 is monoubiquitinated by polycomb repressive complex 1 (PRC1), which cooperates with PRC2 to establish a transcriptionally repressive chromatin environment^25^. Given the known antagonism between H3K36 methylation and polycomb group proteins, it would be interesting to examine whether H2AK119 monoubiquitination suppresses the H3K36 dimethylation activity of NSD2.

**Fig. 3:**
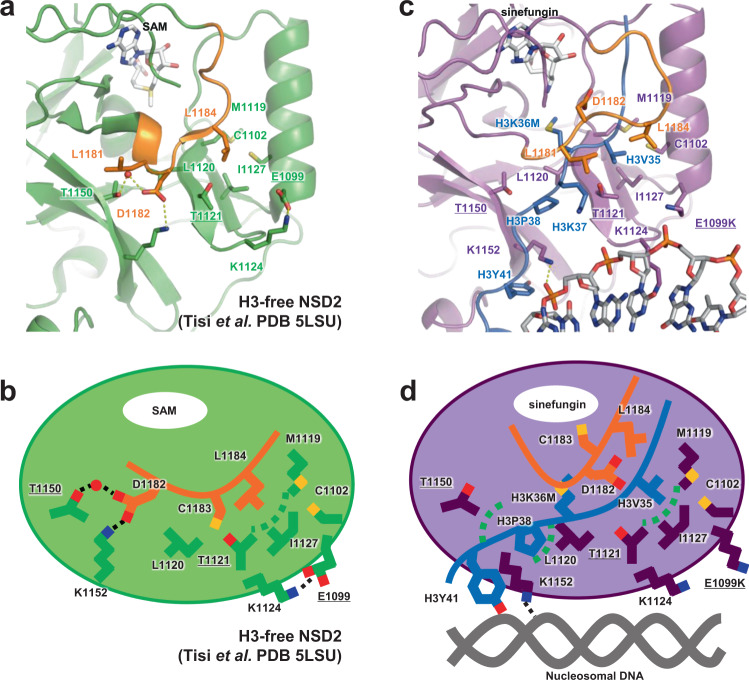
Autoinhibitory loop and oncogenic mutations. Residues whose substitution resulted in increased H3K36 methyltransferase activity are indicated with underlined labels. The autoinhibitory loop is colored orange. A water molecule mediating the interaction with T1150 and D1182 in the H3-free form is shown as a red sphere. **a** Structure of the H3-free NSD2 (PDB ID 5LSU)^18^. **b** A schematic illustration of (**a**). **c** Structure of the NSD2-nucleosome complex. **d** A schematic illustration of (**c**).

H3K36M and adjacent H3 residues are bound to the substrate-binding cleft on the surface of the SET domain of NSD2 (Figs. 2c, 3c, and Supplementary Fig. 6). H3K36M and H3K37 form an intermolecular three-stranded β-sheet structure with M1119-T1121 on one side and Y1179 on the other. The H3T32 carbonyl and hydroxyl groups also form hydrogen bonds with NSD2. Two H3 residues, H3V35 and H3P38, are bound to small hydrophobic patches on the surface of NSD2. The H3V35 side chain interacts with C1102, M1119, T1121, and I1127, while the H3P38 side chain interacts with L1120, T1150, and V1161. The H3K37 side chain protrudes toward the solvent and forms hydrophobic interaction with L1181. The H3K36M side chain is accommodated in the catalytic cavity and surrounded by the side chains of Y1092, L1120, F1177, and Y1179 (Fig. 2d).

Among known H3K36 methyltransferases, NSD proteins and ASH1L have been shown to monomethylate and dimethylate H3K36, while Set2/SETD2 proteins can introduce H3K36 trimethylation. To gain insights into the differences in methylation state specificity among these proteins, we compared our current NSD2-nucleosome structure (Supplementary Fig. 7a) with those of the human SETD2-H3K36M peptide complex (Supplementary Fig. 7b)^22^ and the fungus Set2-nucleosome complex (Supplementary Fig. 7c)^19^ and revealed that in addition to F1177, the other aromatic residues surrounding H3K36M (Y1092 and Y1179 of NSD2) are also conserved. On the other hand, L1120 is replaced with methionine in SETD2 and Set2 (Supplementary Figs. 5, 7). The different residues at this position may regulate methylation state specificity by altering the shape, size, and hydrophobicity of the catalytic cavity.

### Conformational change of the autoinhibitory loop

Fig. 3 shows the structural comparison between H3-free NSD2^18^ and the current structure of NSD2 engaging nucleosomal H3K36. In H3-free NSD2, the autoinhibitory loop (N1180–K1188) adopts a closed conformation, occupying the substrate-binding cleft (Fig. 3a, b). Upon nucleosome binding, the loop adopts an open conformation, making room for the binding of H3K36M and nearby residues (Fig. 3c, d). A similar conformational change of the loop is observed in other H3K36 methyltransferases^13,19,22^

A detailed structural comparison revealed two elements that could be important for such conformational transitions. First, in the H3-free form, D1182 in the autoinhibitory loop forms a network of water-mediated hydrogen bonds and a salt bridge with the side chains of T1150 and K1152, apparently stabilizing the loop conformation (Fig. 3a). Similar interactions are found in the crystal structure of NSD1^26^, implying the functional importance of this conformation shared across NSD family proteins. The T1150A substitution, which is recurrently found in mantle cell lymphoma^12^, could aberrantly increase the catalytic activity of NSD2 by disrupting the interaction with D1182, thereby affecting the conformation of the autoinhibitory loop. In the nucleosome complex, K1152 of NSD2 no longer forms a salt bridge with D1182 and instead interacts with H3Y41 and a DNA phosphate (Figs. 2a, 3c). Thus, K1152 may couple the movement of the autoinhibitory loop with nucleosome binding by switching its binding partner in these forms. This mechanism is consistent with a previous report that the addition of a 41-bp DNA fragment stimulates the catalytic activity of NSD1 and NSD2 on histone octamers^14^.

Second, in the H3-free form, the side chain of L1184 in the autoinhibitory loop binds to the hydrophobic patch formed by C1102, M1119, T1121, and I1127 (Fig. 3a). In the nucleosome complex, the same patch binds to the H3V35 side chain (Fig. 3c). The oncogenic E1099K mutation site is located close to the patch. In the H3-free form, the E1099 side chain forms a salt bridge with K1124, which is located in a loop connecting the two β-strands harboring M1119, T1121, and I1127 (Fig. 3a). In addition to E/K1099, K1124, and K1152, there are several charged residues (such as D1098, D1123, D1125, R1126, and D1158) forming salt bridges with each other around the autoinhibitory loop, which could potentially affect its conformation (see “Discussion”).

### E1099K and T1150A increase the apparent *k*_cat_ value of NSD2

**Fig. 4:**
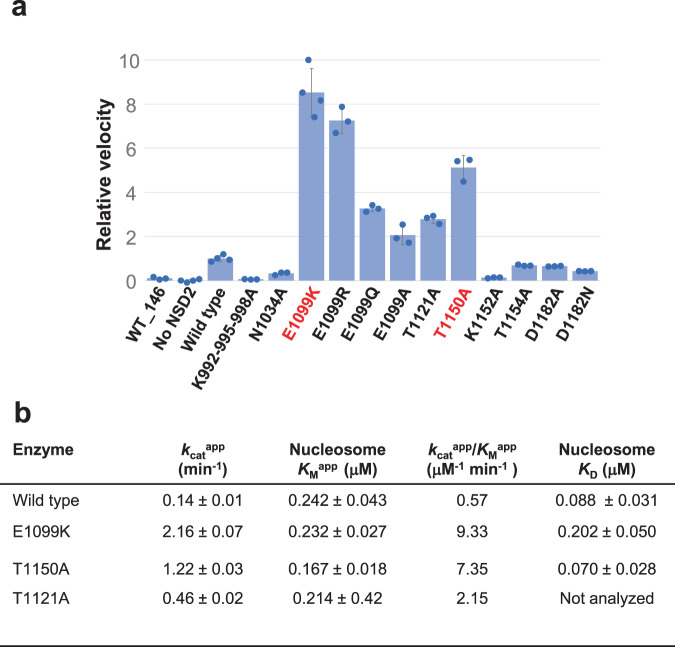
Mutational analyses. **a** Summary of the enzymatic analysis, showing the relative reaction velocity. Data were presented as mean ± standard deviation of independently generated datasets (*n* ≧ 3). Source data are provided as a Source data file. **b** Kinetic values and dissociation constants between the NSD2 proteins and the nucleosome with the 185-bp DNA fragment.

To gain insight into the mechanisms of substrate recognition and their dysregulation by oncogenic mutations, we conducted biochemical analyses. First, we measured the nucleosomal H3K36 methyltransferase activity of NSD2 and its mutants (Fig. 4a) using a commercial kit that couples the production of *S*-adenosyl homocysteine (SAH), a reaction byproduct, to chemiluminescence^27^. NSD2 efficiently methylated the nucleosomes with the 185-bp DNA fragment, but not those with 146-bp DNA fragment, suggesting the importance of its interaction with linker DNA. Accordingly, when the three lysine residues (K992, K995, and K998) that face the linker DNA (Supplementary Fig. 4b) were all mutated to alanines, the mutant lost its catalytic activity. The N1034A, K1152A, and T1154A substitutions reduced catalytic activity, showing the important roles played by the interactions of the residues with the nucleosome (Figs. 2a, 4a, Supplementary Fig. 5). As mentioned above, in the apo structure, D1182 seems to stabilize the closed state of the autoinhibitory loop via its interactions with T1150 and K1152 (Fig. 3a). However, the D1182A mutant showed reduced activity. We speculate that upon engaging nucleosome, the D1182 side chain may interact with H3 (possibly, with the main-chain amido group of H3G34 or H3V35), thus playing a role in substrate binding. Because of a relatively poor density of the autoinhibitory loop in the current structure, we could not build a reliable atomic model for the D1182 side chain.

We next confirmed that the E1099K and T1150A mutants had stronger catalytic activity than the wild-type (Fig. 4a). Additionally, the substitution of E1099 with arginine, glutamine, and alanine resulted in stronger activities (Fig. 4a), suggesting the essential regulatory role of glutamate at this position. Moreover, we found that the T1121A substitution, which alters one of the H3V35-binding patch residues (Fig. 3c), stimulates catalytic activity (Fig. 4a). This structure-guided identification of a novel activating mutation suggests an important role of the H3V35-binding patch in autoinhibition.

Next, we conducted a kinetic analysis to gain further insight into the mechanism of hyperactivation (Fig. 4b, Supplementary Fig. 8). The results showed that the increase in catalytic efficiency (*k*_cat_^app^/*K*_M_^app^) was mainly governed by an increase in the apparent catalytic turnover (*k*_cat_^app^) values of the E1099K, T1150A, and T1121A mutants (~16-, 9-, and 3-fold, respectively). The differences in the *K*_M_^app^ values toward nucleosomes were small among the wild-type and the three mutants. We also measured the affinity between NSD2 and nucleosomes using microscale thermophoresis experiments^28^, which showed that neither E1099K nor T1150A increased nucleosome affinity (Fig. 4b, Supplementary Fig. 9). The E1099K mutant exhibited even weaker affinity, although the positively charged K1099 side chain is located near the nucleosomal DNA. Taken together, the results showed that the oncogenic E1099K and T1150A mutations increased the catalytic turnover but not the nucleosome affinity of NSD2.

We then tested if L1120 is involved in the methylation state specificity. To enhance the signal, we introduced the E1099K background mutation and compare the E1099K single mutant and the E1099K-L1120M double mutant. As shown in Supplementary Fig. 10, the E1099K-L1120M double mutant gave a stronger H3K36 trimethylation signal compared with the E1099K single mutant, with only slight increase in the total SAM consumption. These results show that L1120 is important for the dimethylation specificity of NSD2.

### Impact of E1099K and T1150A on the dynamics of the autoinhibitory loop

To investigate the impact of the E1099K and T1150A mutations on the dynamics of the autoinhibitory loop, we performed MD simulations of the SET domain of the NSD2-SAM complex in the absence of H3. The nucleosome-engaging structure described in this work is highly similar to that of apo-NSD2 except for the autoinhibitory loop (Supplementary Fig. 11) and was used as the template to prepare the initial structures. We added the N-terminal helix (residues 973–985) from the apo-NSD2 structure, replaced sinefungin with SAM, and introduced appropriate mutations. We then performed runs on the wild-type and three NSD2 mutants (E1099K, T1150A, and the E1099K-T1150A double mutant, Supplementary Table 2) and analyzed the trajectories obtained from three independent 500-ns runs for each system. The residue-wise contact map (Supplementary Fig. 12) and the time-dependent developments of the secondary structure elements (Supplementary Fig. 13) showed that in all systems, the local structures and the secondary elements were maintained throughout the simulation, except in the following two regions. The N-terminal helix, whose density was not observed in this study, was sometimes lost in the simulation. In all of the systems, the conformation of the autoinhibitory loop was flexible during the simulation, suggesting that the H3-engaging conformation is not stable in the absence of H3 (Supplementary Movie 1).

**Fig. 5:**
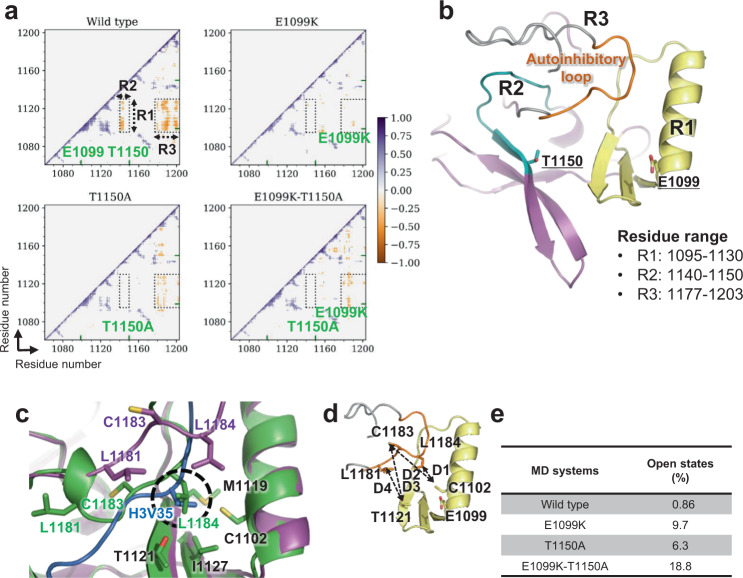
Opening and closing of the autoinhibitory loop. **a** 2D plots of the cross-correlation of residue-wise fluctuations in the NSD2 wild-type, E1099K, T1150A, and double mutant E1099K-T1150A proteins. All the correlation coefficients between −0.3 and 0.3 were taken as zero to visualize the significantly correlated residues. The regions labeled R1 (residues 1095–1130), R2 (residues 1140–1150), and R3 (post-SET loop) exhibit a remarkable difference between the wild-type and mutants. The mutation sites are denoted by green ticks on the axis. **b** The regions R1 (pale yellow), R2 (teal), R3 (gray), the autoinhibitory loop (orange) in R3 and the mutation sites (stick view), are shown and labeled on the NSD2 SET domain structure. **c** Superimposition of H3-free NSD2 (green) and nucleosome-bound NSD2 (magenta). H3 is shown in marine blue. The overlapping hydrophobic cavity occupied by the L1184 residue in substrate-free NSD2 and H3V35 is circled in black. **d** Definition of four distance-based locks (D1: L1184–C1102, D2: C1183–C1102, D3: C1183–T1121, and D4: L1181–T1121), as represented by dashed lines in the SET domain structure. The autoinhibitory loop is shown in orange, and residues are labeled. **e** Table showing the percentage of open autoinhibitory loop conformations observed under different conditions.

We first calculated the dynamic cross-correlation of the SET-domain residue-wise fluctuations to capture the correlated motions among the residues. In the wild-type, we observed anti-correlated motions between residues 1095–1130 (region R1) and 1140–1150 (region R2) as well as R1 and residues 1177–1203 (region R3) (Fig. 5a, b). R1 contained E1099 and residues forming the H3V35- and H3P38-binding hydrophobic patches, whereas R2 and R3 contained T1150 and the autoinhibitory loop, respectively. In all three mutants, the anti-correlated motions between R1–R2 and R1–R3 disappeared (Fig. 5a), indicating that the movement of R2 and R3 became independent of R1.

The independent movement of R3 suggested that the autoinhibitory loop might tend to adopt conformations that allow histone H3 binding in the mutants more often. To test this hypothesis, we introduced four distance-based measures (hereafter referred to D1, D2, D3, and D4 locks) representing the presence or absence of hydrophobic interactions between residues in the autoinhibitory loop (L1181, C1183, and L1184) and those in the H3V35-binding patch (C1102 and T1121) (Fig. 5c, d). The time development of the four distances is shown in Supplementary Fig. 14. We defined the autoinhibitory loop as “open” when all four locks were released and “closed” otherwise. The probabilities of open states of the loop and individual locks are shown in Fig. 5e and Supplementary Tables 3 and 4. In the wild-type, the autoinhibitory loop was open for only 0.86% of the simulation time, indicating that the loop almost always sits in the substrate-binding cleft. In the E1099K, T1150A, and E1099K-T1150A mutants, open states appeared far more frequently (9.7%, 6.3%, and 18.8%, respectively). A detailed examination of the MD trajectories revealed a novel mode of interaction between the autoinhibitory loop and the hydrophobic patches (Supplementary Fig. 15). In the wild-type, the L1181 side chain often forms hydrophobic interactions with T1121 and/or T1150, the two threonine residues whose substitution leads to aberrant enzymatic activation. These interactions occurred less often in the three mutants, as indicated by the frequency of the operational D4 lock (94.9%, 26.3%, 78.9%, and 56.2% for the wild-type, E1099K, T1150A, and E1099K-T1150A, respectively). Moreover, an entropy calculation showed that the states of these four locks are more evenly distributed in the mutants than in the wild-type, suggesting that the loop changes its state more frequently in the mutants (Supplementary Table 4). Collectively, our MD analysis suggests that the E1099K and T1150A mutations affect the dynamics of the autoinhibitory loop, causing NSD2 to adopt open states more often to accommodate the H3 tail in the substrate-binding cleft.

## Discussion

Our structural and kinetic analyses show that the oncogenic E1099K and T1150A mutants increase the catalytic turnover of NSD2, leading to its hyperactivation. Moreover, based on the results of MD simulation, we propose a working model of NSD2 in which the autoinhibitory loop conformation regulates its catalytic activity and the oncogenic E1099K and T1150A mutations aberrantly disturb autoinhibition. In the H3-free form, the autoinhibitory loop dynamically moves and exhibits multiple conformations while remaining bound to the H3V35- and H3P38-binding patches via hydrophobic interactions involving L1181, C1183, and L1184 and hydrophilic interactions involving D1182. Consequently, the substrate-binding cleft is almost always occupied by the autoinhibitory loop, leaving no room for H3 binding. Upon nucleosome binding, the conformation of the autoinhibitory loop changes, allowing H3V35 and H3P38 to be accommodated in hydrophobic patches and enabling precise positioning of the H3K36 side chain for methyl group transfer. This transition is partially triggered by the binding of H3Y41 and nucleosomal DNA to K1152, which is incompatible with its interaction with D1182. The E1099K and T1150A mutants affect the conformational dynamics of the autoinhibitory loop, leading to an increased tendency to adopt open states.

There could be several possible mechanisms by which the E1099K and T1150A mutations affect the conformation of the autoinhibitory loop. In the MD trajectories, a cluster of charged residues surrounding the autoinhibitory loop forms a network of salt bridges (Supplementary Fig. 16). In the trajectories of the E1099K and E1099K-T1150A mutants, the E1099K mutation remodels the network by forming a novel salt bridge with D1125, which is accompanied by decreased salt bridge formation between D1123 and K1152 (Supplementary Fig. 16c, e). Similarly, in the T1150A mutant, the salt bridge between D1123 and K1152 is reduced (Supplementary Fig. 16d). The salt bridge between D1123 and K1152 connects the two β-sheets possessing H3V35- and H3P38-binding patches, and interestingly, the autoinhibitory loop tends to assume an open state when this salt bridge is broken (Supplementary Fig. 16f). These observations suggest that the E1099K and T1150A mutations may remodel the network of salt bridges formed by the charged residues surrounding the autoinhibitory loop, thus affecting its dynamics. This hypothesis is consistent with our biochemical assay showing that the substitution of E1099 with neutral amino acids (alanine and glutamine) has a smaller effect than substitution with basic amino acids (lysine and arginine). Alternatively, the T1150A mutant may lose the ability to maintain the loop in a closed state because of the lack of interaction between T1150 and D1182 or L1181. Further studies, such as additional mutant analyses and MD studies with nucleosomal substrates, will be required to test our hypotheses and elucidate the detailed molecular mechanisms of these oncogenic mutants.

While this manuscript was being completed, Li et al. reported the structures of NSD2 and NSD3 bound to nucleosome at resolutions of 3.15–3.75 Å^13^. The overall structures reported here and by Li et al. are very similar, showing that our fusion construct improved the structural resolution by stabilizing the complex without introducing artifacts. The mechanisms proposed by Li et al. differ from ours regarding the aberrant hyperactivation of NSD proteins by oncogenic mutations. E1099K has been proposed to increase the electrostatic interactions between NSD2 and the nucleosome, thereby enhancing its catalytic activity. However, the kinetic and affinity analyses conducted by both Li et al. and our group showed that E1099K does not significantly increase nucleosome affinity. Li *et al*. also showed that the T1232A mutation of NSD3 (corresponding to the T1150A mutation of NSD2) resulted in a structural shift in the H3P38-H39 region by ~1.4 Å, forming newly created hydrogen bonds between NSD3 and H3. It is not clear if the same mechanism applies to NSD2. When the current NSD2-nucleosome complex (bearing E1099K) was compared with the NSD2-nucleosome complex by Li et al. (bearing E1099K and T1150A) via the superposition of their NSD2 molecules, the coordinate shifts in the Cα atoms of H3P38 and H3H39 were relatively small (0.5 and 0.3 Å, respectively), suggesting that the T1150A mutation may not significantly affect the conformation of the H3P38-K39 region (Supplementary Fig. 17).

We propose that under normal conditions, nucleosomal DNA acts as a positive allosteric regulator by interacting with K1152 and thereby influencing the conformational dynamics of the autoinhibitory loop. This is reminiscent of the regulatory mechanism of ASH1L, another H3K36 dimethyltransferase^29,30^. By itself, ASH1L has weak catalytic activity, which is stimulated by its binding partner, MRG15. Structural analyses revealed that MRG15 binding indirectly influences the dynamics of the autoinhibitory loop. Thus, influencing the dynamics of the autoinhibitory loop may be a general mechanism regulating the activity of several H3K36 methyltransferases. On the other hand, other families of histone methyltransferases, such as PRC2 and Set1/MLL family proteins, employ different mechanisms for allosteric activation^31^.

Our study also provides structural insights into how the H3K36 methylation state can be regulated by nucleosome structure. The linker histone H1 has been shown to inhibit the methyltransferase activity of NSD2 toward chromatin^15,16^. Loss-of-function mutations in genes encoding H1 isoforms are highly recurrent in B cell lymphomas and cause a genome-wide increase in H3K36me2^16,32^, demonstrating the critical importance of H1 for antagonizing NSD proteins for normal gene regulation. H1 binds to the nucleosome dyad and contacts both linker DNAs, stabilizing a compact conformation^33,34^ (Supplementary Fig. 18f). Our structure shows that NSD2 binds the nucleosome with partially unwrapped DNA. H1 may thus inhibit the activity of NSD2 by hindering the unwrapping of nucleosomal DNA and, hence, its access to H3K36. On the other hand, several chromatin factors, such as ATP-dependent chromatin remodelers^35^, so-called “pioneer transcription factors” that can recognize chromatinized DNA elements^36^, and RNA polymerase II^37^, are known to affect the conformation of nucleosomal DNA. Interestingly, the binding of such factors to nucleosomes results in the partial unwrapping of nucleosomal DNA, similar to what is observed for the NSD2 complex (Supplementary Fig. 18a–e). It may be speculated that chromatin factors and H3K36 methyltransferases work cooperatively such that H3K36 exposure caused by other factors (such as chromatin transcription by RNA polymerase II) facilitates efficient methylation. In this case, H3K36 is well positioned to act as a memory mark of changes in the nucleosomal structure, as conformational changes in nucleosomal DNA affect its accessibility.

## Methods

### DNA preparation

We prepared the 146-bp and 185-bp DNA fragments using different strategies. The 146-bp DNA fragment was excised by EcoRV from a plasmid DNA sequence containing 14 copies of the Widom 601 DNA sequence. The excised DNA was applied to HiTrap Q column chromatography (GE Healthcare), eluted with a NaCl gradient (0–1000 mM) in 50 mM HEPES-Na pH 7.5, flash-frozen with liquid nitrogen (LN_2_), and stored at −80 °C. The 185-bp DNA fragment was generated using nested PCR amplification. Initially, a 282-bp DNA fragment containing the Widom 601 DNA sequence was amplified using pGEM-3Z-601^38^ as a template and 282 F and 282 R as primers (Supplementary Table 5) and was subsequently purified by acrylamide gel electrophoresis. The 185-bp DNA fragment was amplified using the 282-bp DNA fragment as the template and 185F and 185R (Supplementary Table 5) as primers. The amplified 185-bp DNA fragment was applied to HiTrap Q column chromatography, eluted with a NaCl gradient (0–1500 mM) in 20 mM Tris-HCl pH 8.0 and 1 mM EDTA, flash-frozen with LN_2_, and stored at −80 °C.

### Protein preparation

The sequences of the protein-coding regions are shown in Supplementary Table 6. Human histone H2A was bacterially expressed as a His6- and SUMOstar-tagged protein, and the protein was then isolated from inclusion bodies under denaturing conditions (20 mM Tris-HCl, pH 7.5, 7 M guanidine hydrochloride, 500 mM NaCl, 1 mM DTT). The supernatant was applied to Ni Sepharose High Performance (GE Healthcare), washed with 20 mM Tris-HCl, pH 7.5, 7 M urea, and 1 M NH_4_Cl, then eluted with 20 mM Tris-HCl, pH 7.5, 7 M urea, 1 M NH_4_Cl, and 500 mM imidazole, and dialyzed against buffer containing 20 mM Tris-HCl, pH 7.5, 150 mM NaCl, and 0.5 mM DTT. The His6-SUMOstar-tag was removed by SUMOstar protease digestion. The resulting histone H2A protein was applied to HiTrap SP column (GE Healthcare), eluted with a NaCl gradient (400–1500 mM) in 20 mM HEPES-Na, pH 7.5, and 1 mM DTT, flash-frozen with LN_2_, and stored at −80 °C.

Human histone H2B was bacterially expressed as a His6-tagged protein and isolated from inclusion bodies under denaturing conditions (20 mM Tris-HCl pH 7.5, 7 M guanidine hydrochloride, 500 mM NaCl, 1 mM DTT). The supernatant was applied to cOmplete His-Tag Purification Resin (Roche), washed with 20 mM Tris-HCl, pH 7.5, 7 M urea, 1 M NH_4_Cl, and 1 mM DTT, then eluted with 20 mM Tris-HCl pH 7.5, 7 M urea, 1 M NH_4_Cl, and 500 mM imidazole, and dialyzed against buffer containing 20 mM Tris-HCl, pH 7.5, 100 mM NaCl, and 1 mM DTT. The His6-tag was removed by thrombin digestion. The resulting histone H2B protein was applied to HiTrap SP column, eluted with a NaCl gradient (100–2000 mM) in 10 mM Tris-HCl, pH 7.5, flash-frozen with LN_2_, and stored at −80 °C.

His6 and SUMOstar-tagged human histone H4 was bacterially coexpressed with human histone H3 and purified under nondenaturing conditions. Cells coexpressing H3 and H4 were suspended in buffer containing 40 mM K_2_HPO_4_, 10 mM KH_2_PO_4_, 20 mM imidazole, 3 M NaCl, 1 mM DTT, 0.1 mM PMSF, and 0.5% Triton X-100 and then sonicated. The supernatant was applied to HisTrap column (GE Healthcare), eluted with 20 mM Tris-HCl, pH 7.5, 7 M urea, 1 M NH_4_Cl, and 500 mM imidazole. The His6-SUMOstar-tag was removed by SUMOstar protease digestion. The resulting histone H3/H4 complex was dialyzed against dialysis buffer (10 mM Tris-HCl, pH 7.5, 250 mM NaCl, 1 mM DTT). The dialyzed protein was applied to HiTrap SP column, eluted with a NaCl gradient (250–1500 mM) in 10 mM Tris-HCl, pH 7.5 and 1 mM DTT, flash-frozen with LN_2_, and stored at −80 °C.

His6 and SUMOstar-tagged human NSD2-E1099K were fused to H4 and bacterially coexpressed with human histone H3 possessing a K36M substitution. The complex between NSD2-E1099K-H4 and H3 was purified in a way similar to the H4-H3 complex; however, the His6-SUMOstar tag was not removed.

Wild-type and mutant NSD2 proteins (973–1226) containing Twin-Strep-tag and His6-tag at the N-terminus were bacterially expressed. Cells were suspended in buffer containing 40 mM K_2_HPO_4_, 10 mM KH_2_PO_4_, 500 mM NaCl, 1 mM DTT, 0.1 mM PMSF, and 0.5% Triton X-100 and then sonicated. The supernatant was applied to StrepTrap column (GE Healthcare), eluted with 40 mM K_2_HPO_4_, 10 mM KH_2_PO_4_, 500 mM NaCl, 1 mM DTT, and 2.5 mM desthiobiotin, flash-frozen with LN_2_, and stored at −80 °C. The primers to create plasmids expressing mutant NSD2 proteins are shown in Supplementary Table 5.

### Histone octamer preparation

We incubated histones H2A, H2B, and H3/H4 at a molar ratio of 1.2:1.2:1 in unfolding buffer (6 M guanidine hydrochloride, 4 mM HEPES-Na pH 7.5, 200 mM NaCl, 5 mM DTT) at 4 °C for 1 h, followed by dialysis against refolding buffer (10 mM Tris-HCl, pH 7.5, 2 M NaCl, 1 mM EDTA, 5 mM 2-mercaptoethanol) to reconstitute the histone octamer. The octamer was eventually purified from the unincorporated components using a Superdex 200 pg 26/60 column (GE Healthcare) equilibrated with buffer containing 10 mM Tris-HCl pH 7.5, 2 M NaCl, 1 mM EDTA, and 5 mM 2-mercaptoethanol, flash-frozen with LN_2_, and stored at −80 °C.

### Nucleosome reconstitution

To reconstitute nucleosomes for enzymatic and interaction analyses, we mixed the 185-bp (final 6.1 μM) or 146-bp (final 5.6 μM) DNA fragment with a histone octamer at a molar ratio of ~1:1.1 and then performed dialysis against 125 mL of 10 mM HEPES-Na pH 7.5, 2 M KCl, and 1 mM DTT for 1 h. Thereafter, 875 mL of 10 mM HEPES-Na pH 7.5 and 1 mM DTT was gradually added to facilitate nucleosome reconstitution. The nucleosome samples were further dialyzed against 10 mM HEPES-Na pH 7.5, 50 mM KCl, and 1 mM DTT. The centrifuged supernatant was eventually concentrated and stored at 4 °C.

To prepare the NSD2-nucleosome complex for cryo-EM analysis, we mixed the 185-bp DNA fragment (final 2.4 μM) with H2A, H2B, and the complex between NSD2 E1099K-H4 and H3K36M at a molar ratio of ~1:2.6:2.6:2.6 and dialyzed the mixture for 1 h against 125 mL of buffer containing 10 mM HEPES-Na pH 7.5, 2 M KCl, and 1 mM DTT. Thereafter, 875 mL of 10 mM HEPES-Na (pH 7.5) and 1 mM DTT was gradually added to facilitate the reconstitution of nucleosomes. The NSD2-nucleosome complex was further dialyzed against 10 mM HEPES-Na pH 7.5, 1 mM DTT, and 25 mM or 50 mM KCl. The centrifuged supernatant was concentrated, and a sinefungin solution (25 mM) was added (final 1.3 mM) before cryo-EM data acquisition.

### Sample vitrification and cryo-EM data acquisition

The reconstituted NSD2-nucleosome complex was applied to a freshly glow-discharged Quantifoil holey carbon grid (R1.2/1.3, 300 mesh, Cu/Rh grid for the sample with 25 mM KCl, and Au grid for the sample with 50 mM KCl), subjected to blotting for 4 s at 4 °C in 100% humidity, and plunge-frozen in liquid ethane using a Vitrobot Mark IV (Thermo Fisher Scientific). Grid images were obtained using a 300 kV Titan Krios G3i microscope (Thermo Fisher Scientific) equipped with a K3 direct electron detector (Gatan) installed at the University of Tokyo, Japan. Data sets were acquired with SerialEM software, with a defocus range of −0.8 to −1.6 μm. Data acquisition statistics are shown in Supplementary Table 1.

### Image processing and model building

Movie stacks were corrected for drift- and beam-induced motion using MotionCor2^39^, and the CTF parameters were estimated using GCTF^40^. Particles were automatically picked using RELION-3.1^41^ and then used for two-dimensional (2D) classification, ab initio reconstruction, and hetero refinement with cryoSPARC^42^. The particles that converged into the NSD2-nucleosome complex class were exported to RELION-3.1 and used for focused three-dimensional (3D) classification with a mask covering only NSD2. The particles that converged to the class with a well-resolved NSD2 density were used for final 3D refinement with RELION-3.1. The resolution was estimated based on the gold-standard Fourier shell correlation (FSC) curve according to the 0.143 criterion. The atomic models of H3-free NSD2 (PDB 5LSU) and nucleosome (PDB 1KX5) were fit into the density using UCSF Chimera^43^ and then manually modified using Coot^44^. Real-space refinement was performed using Phenix^45^.

### Methyltransferase assay

For a simple comparison of the initial reaction rate, wild-type or mutant NSD2 (128 nM) and nucleosomes (4 μM) were used (Fig. 4a). For kinetic analysis, wild-type NSD2 (256 nM), the E1099K mutant (16 nM) or the T1150A mutant (32 nM), and a series of twofold diluted nucleosomes (from 31.25 nM to 4 μM) were used (Fig. 4b, Supplementary Fig. 8). The NSD2 proteins, SAM (30 μM), and nucleosomes were mixed in reaction buffer (2.5 mM HEPES-Na pH 7.5, 10 mM Tris-HCl pH 9.5, 2.5 mM NaCl, 12.5 mM KCl, 1 mM TCEP, and 0.01% Tween 20), and the mixture was incubated at 30 °C. Approximately 4 μL of each reaction mixture was collected at 2, 4, 6, and 8 min and quenched by adding 1 μL of 0.5% TFA. Methyltransferase activity was evaluated using an MTase-Glo Methyltransferase Assay Kit (Promega). The luminescent signal corresponding to SAH production was measured three times using Centro LB 960 (Berthold Technologies) in a white half-area 96-well plate. The measured luminescent signal was converted to represent the amount of SAM utilized with an SAH standard curve. The initial rate of each reaction was determined using a linear regression fit of the data. Each reaction was run in triplicate. Kinetic parameters were derived by fitting the values to the Michaelis–Menten model using KaleidaGraph 4.5.3 software.

### Microscale thermophoresis

The microscale thermophoresis (MST) assay was performed using a Monolith NT.115 instrument (NanoTemper Technologies). For the MST assay, His-tagged NSD2 proteins (50 nM) labeled with RED-Tris-NTA and a series of twofold-diluted nucleosome or DNA (from 0.31 nM to 10 μM) were incubated in the binding buffer (20 mM HEPES-Na pH 7.5 150 mM KCl, 5% glycerol, 0.05% Tween 20, 1 mM DTT, and 1 mM sinefungin) for 30 min at room temperature and centrifuged at 22,140 × *g* for 5 min. Premium capillaries were filled with the samples to obtain measurements with an extinction power of 60% and medium MST power at 25 °C. Thermophoresis data were analyzed using MO. Affinity Analysis software ver. 2.3 (NanoTemper Technologies).

### Trimethyltransferase assay

For H3K36 trimethyltransferase assay, the E1099K single mutant and E1099K-L1120M double mutant were used at 128 nM. The mutant NSD2 proteins, SAM (30 μM), and nucleosomes (4 μM) were mixed in the reaction buffer same as above and were incubated at 30 °C for 3 h. 6 μL of the reaction mixture was subjected to SDS-PAGE (8% acrylamide) using Rapid Running Buffer Solution (nacalai tesque) and then transferred to Immobilon-P PVDF membranes (Merck). The membranes were blocked for 1 h with 3% BSA in TBS-T (100 mM Tris (pH 7.2), 150 mM NaCl, 0.05% Tween-20). Membranes were then incubated with primary antibody against H3K36me3 (#4909, Cell Signaling Technology) diluted in Can Get Signal solution 1 (TOYOBO) for 1 h (1:1000), and secondary antibody (sc-2004, Santa Cruz Biotechnology) diluted in Can Get Signal solution 2 for 1 h (1:2000) at room temperature. Following extensive washing, protein bands were visualized with ECL Prime Western Blotting Detection Reagent (cytiva) and band densities were analyzed with LAS-3000 mini luminescent image analyzer and Multi Gauge version 3.0 software (FUJIFILM).

### Molecular dynamics simulations

The NSD2 E1099K structure (residues 986–1203 constituting two Zn atoms in the AWS domain, one Zn atom, and SAM in the SET domain) was extracted from the cryo-EM-based nucleosome-bound NSD2 E1099K complex reported in this study by deleting the nucleosome. The N-terminal helix region (residues 973–985) from the crystal structure^18^ (PDB ID: 5LSU) was added to the extracted NSD2 structure. In the combined structure, the L975 and L978 residues of the N-terminus were mutated back to the original sequence residues (Q975 and K1073, respectively) to obtain the initial structure of NSD2 E1099K with an open autoinhibitory loop conformation. Subsequently, the initial structures of wild-type NSD2 and the T1150A and E1099K-T1150A mutants were obtained by introducing appropriate mutations. All mutations were introduced using the rotamer library in UCSF Chimera^43,46^. Thus, we performed MD simulations of the NSD2 wild-type and three mutants, namely, E1099K, T1150A, and E1099K-T1150A (Supplementary Table 2).

All simulations were performed using the AMBER package with the ff14SB force field for proteins and improved parameters for SAM^47–49^. The parameters of the Zn ions coordinated with C1144, C1191, C1193, and C1198 were obtained from the zinc AMBER force field (ZAFF)^50^. The parameters of the two Zn ions in the AWS domain coordinated with seven cysteine residues were generated using the MCPB.py program^51^. Geometry optimization and RESP charge (Merz–Kollman scheme) calculations were performed at the B3LYP/6-31G* level of theory in Gaussian16^52–54^. While preparing the parameters of the proteins, no specific protonation state treatments were performed for any residues in this study except for the cysteines coordinated with the Zn atoms in the SET and AWS domains. All the cysteines coordinating with Zn atoms were deprotonated.

Each system was solvated and neutralized in a cubical box containing a 0.150 M NaCl and TIP3P water model^55^ solution with a padding distance of 13.5 Å. Energy minimization involved both steepest-descent and conjugate-gradient algorithms. System equilibration was performed in three successive steps of 500 ps each. First, heating to 300 K in NVT was performed, followed by equilibration under NPT at a temperature of 300 K and a pressure of 1 bar. A positional restraint of 100 kcal mol^-1^ Å^−2^ was applied to the heavy atoms of the solute during the first two cycles of equilibration. The temperature of 300 K and pressure of 1 bar were maintained using the Langevin dynamics algorithm (collision frequency γ = 2.0 and coupling constant = 1.0 ps) and the Berendsen barostat (pressure relaxation time = 1.0 ps). Particle mesh Ewald (PME) was used to calculate long-range electrostatic interactions^56^, and the bonds associated with hydrogen atoms were constrained using the SHAKE algorithm^57^. Finally, three independent 500-ns runs were performed for each system. All analyses for the last 400-ns simulation of each independent run of the trajectories were performed using the CPPTRAJ package^58^.

### Figure preparation

Structural figures were created using PyMOL (Schrödinger, LLC) and UCSF ChimeraX^59^. Sequence alignment figures were created using ESPript^60^. Both trajectory visualization and movie creation were accomplished using VMD^61,62^.

### Reporting summary

Further information on research design is available in the Nature Research Reporting Summary linked to this article.

## Supplementary information

Supplementary Information

Description of Additional Supplementary Files

Supplementary Movie 1

Reporting summary

## Data Availability

The density map and model coordinates have been deposited to Electron Microscopy Data Bank (accession number EMD-31015) and Protein Data Bank (accession number 7E8D), respectively. Source data are provided with this paper.

## Source data

Source data
