# Supplementary material for: Structural basis of the regulation of the normal and oncogenic methylation of nucleosomal histone H3 Lys36 by NSD2
Source: Nat Commun. 2021 Nov 15;12:6605. doi: 10.1038/s41467-021-26913-5 (PMC8593083; doi:10.1038/s41467-021-26913-5)
Supplement: Supplementary file 1 — Supplementary Information [file 41467_2021_26913_MOESM1_ESM.pdf]

Supplementary Information

Structural basis of the regulation of the normal and oncogenic methylation of nucleosomal histone H3 Lys36 by NSD2

Ko Sato[1#], Amarjeet Kumar[2], Keisuke Hamada[1], Chikako Okada[1], Asako Oguni[1], Ayumi Machiyama[1], Shun Sakuraba[2], Tomohiro Nishizawa[3,4], Osamu Nureki[3], Hidetoshi Kono[2], Kazuhiro Ogata[1*], and Toru Sengoku[1#*]

[1]Department of Biochemistry, Yokohama City University Graduate School of Medicine, 3-9 Fukuura, Kanazawa-ku, Yokohama, Kanagawa 236-0004, Japan

[2]Institute for Quantum Life Science, National Institutes for Quantum Science and Technology, 8-1-7 Umemidai, Kizugawa, Kyoto 619-0215, Japan

[3]Department of Biological Sciences, Graduate School of Science, The University of Tokyo, 7-3-1 Hongo, Bunkyo-ku, Tokyo 113-0033, Japan

[4]Current address: Graduate School of Medical Life Science, Yokohama City University, 1-7-29 Suehiro-cho, Tsurumi-ku, Yokohama, Kanagawa 230-0045, Japan

# Contributed equally

* To whom correspondence should be addressed:

ogata@yokohama-cu.ac.jp and tsengoku@yokohama-cu.ac.jp

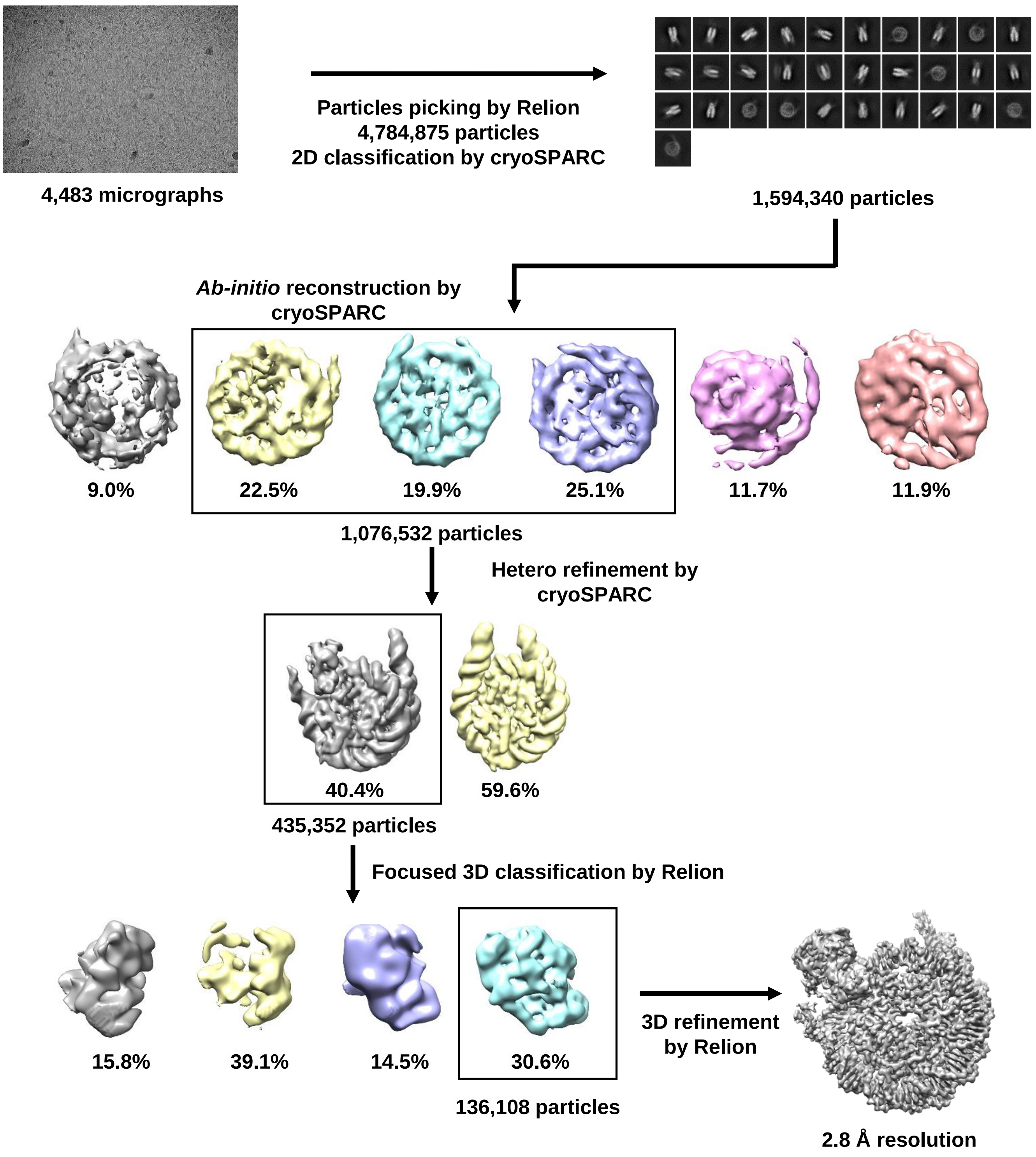

Supplementary Figure 1 Cryo-EM 3D reconstruction and refinement.

a

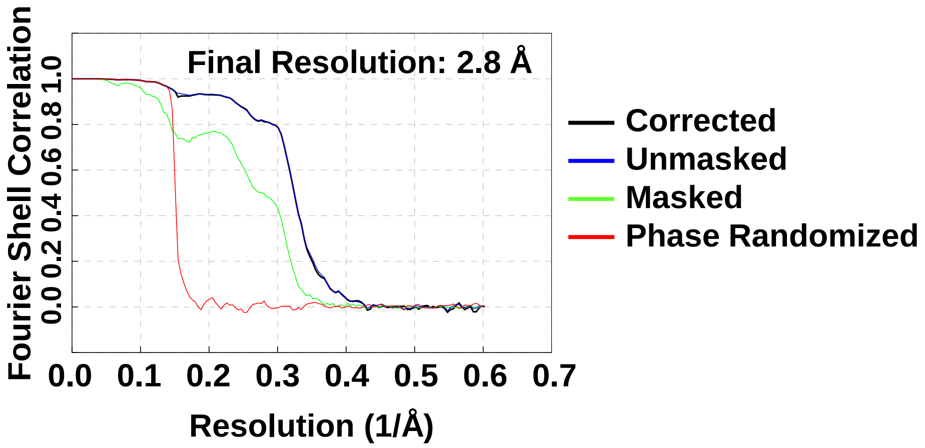

b

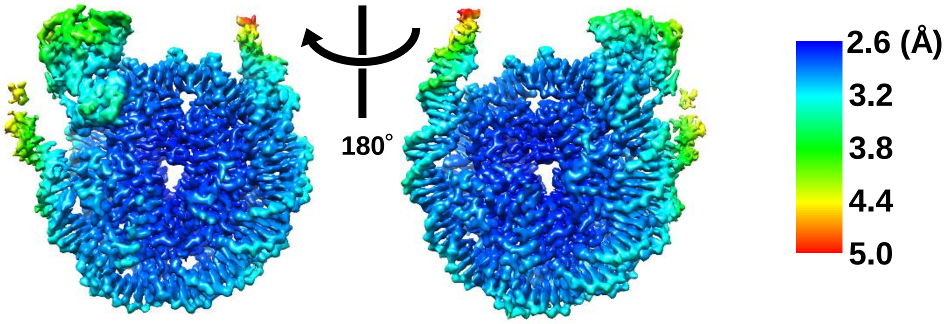

Supplementary Figure 2 Global and local resolution of the 3D reconstituted map. a, Fourier shell correlation (FSC) curves of the 3D reconstruction. The resolution was estimated based on the FSC curve according to the 0.143 criterion. **b**, Local resolution map of the NSD2-nucleosome complex.

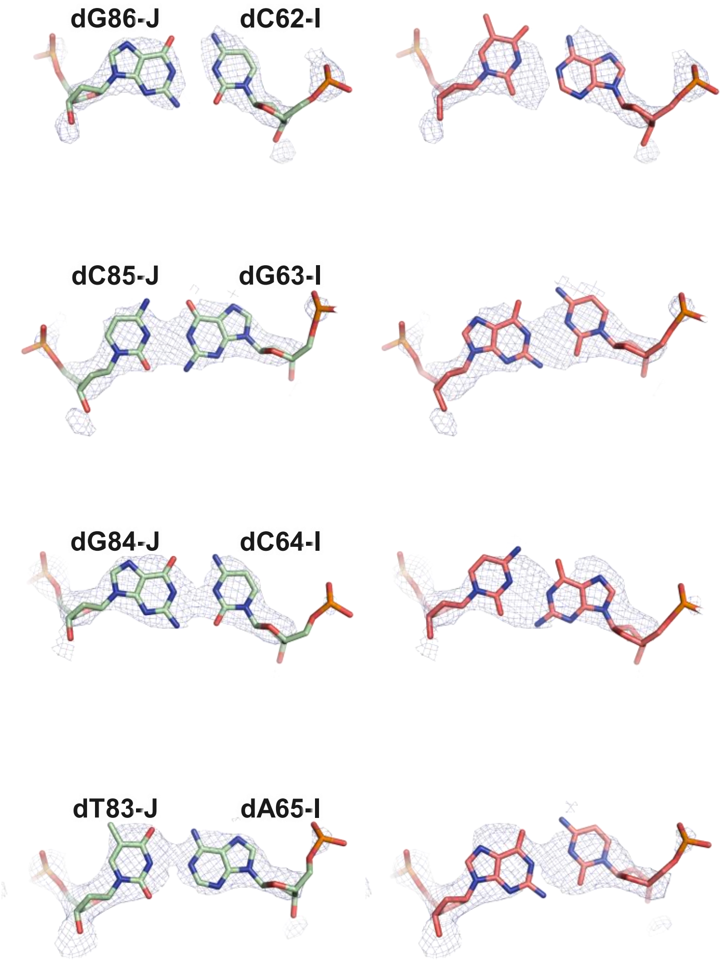

Supplementary Figure 3 The density of base pairs at several positions shows that NSD2 preferentially binds to the DNA at the end of the 601 sequence known to be unwrapped more easily. The density of nucleosomal DNA near SHL +0.5 is shown. Between the two possible orientations of the nucleosomal DNA, one (left, adopted in the current structure) better fits the density of purine-pyrimidine base pairs at these positions than the other (right).

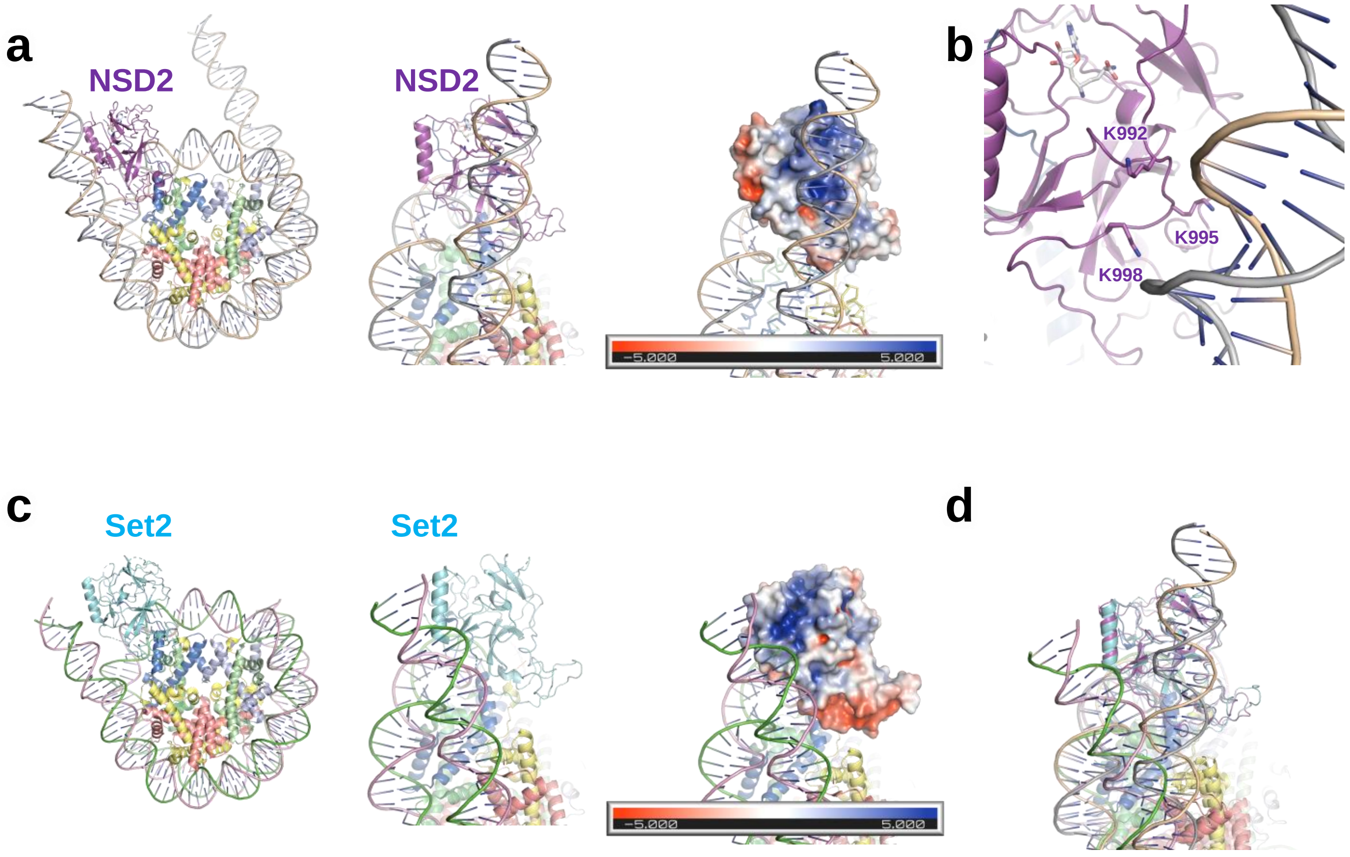

Supplementary Figure 4 Structural comparison between NSD2 and yeast Set2. a, Structure of the NSD2-nucleosome complex from two views (left) and electrostatic potential mapped on the surface of NSD2 (right). **b**, A close-up view of the three lysine residues that interact with nucleosomal DNA in the NSD2-nucleosome complex. **c**, Structure of the Set2-nucleosome complex (PDB ID 6NZO) from two views (left) and electrostatic potential mapped on the surface of Set2 (right). **d**, Superposition of the NSD2-nucleosome and Set2-nucleosome structures.

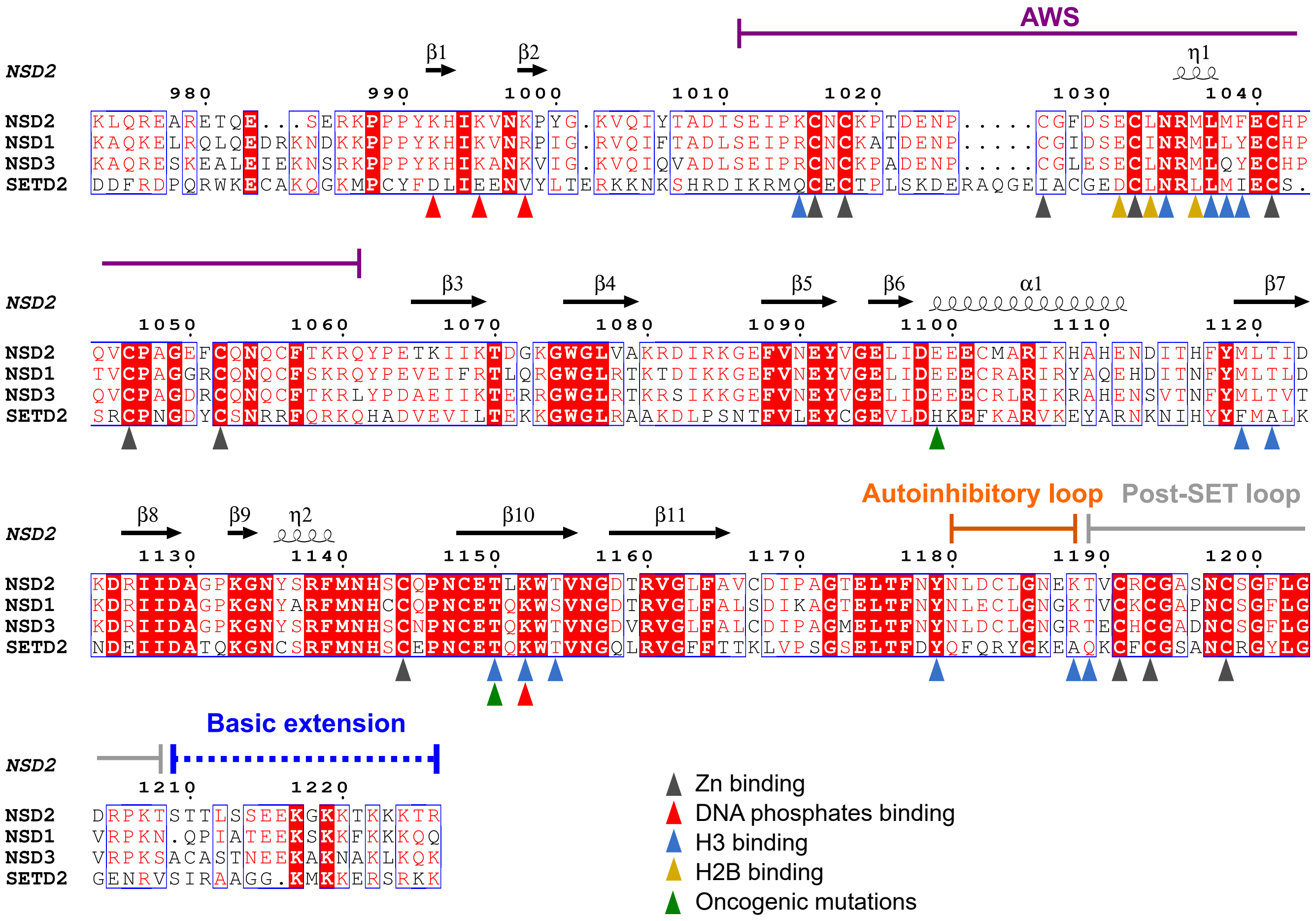

Supplementary Figure 5 Sequence alignment of human NSD1, NSD2, NSD3, and SETD2.

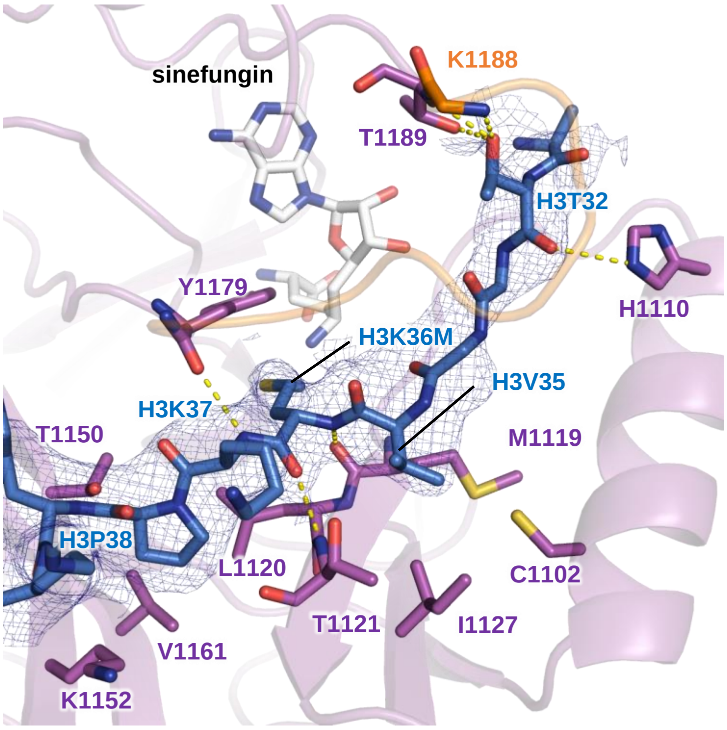

Supplementary Figure 6 The H3K36-binding cavity with the cryo-EM density at 5σ shown as blue mesh.

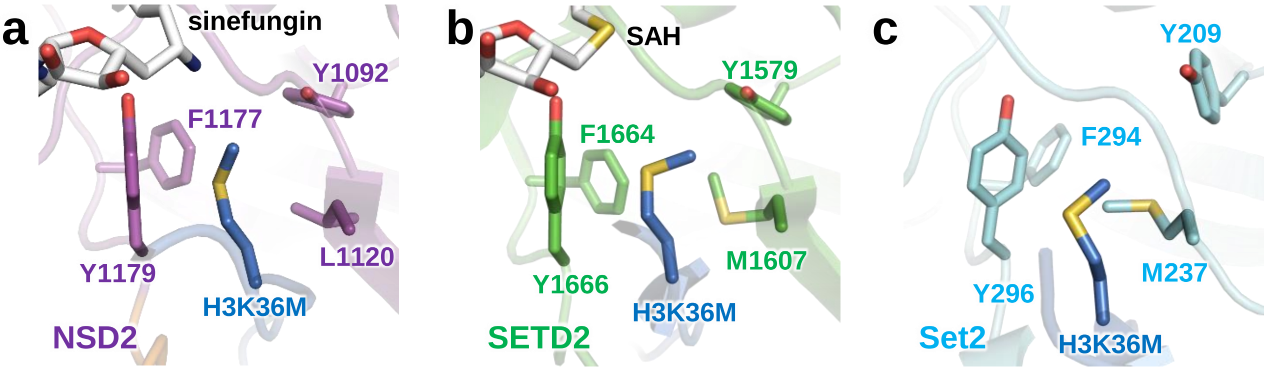

Supplementary Figure 7 Structural comparison of the H3K36-binding cavities of NSD2, human SETD2, and yeast Set2. a, NSD2 (the current structure). **b**, SETD2 (PDB ID 5JJY). **c**, Set2 (PDB ID 6NZO).

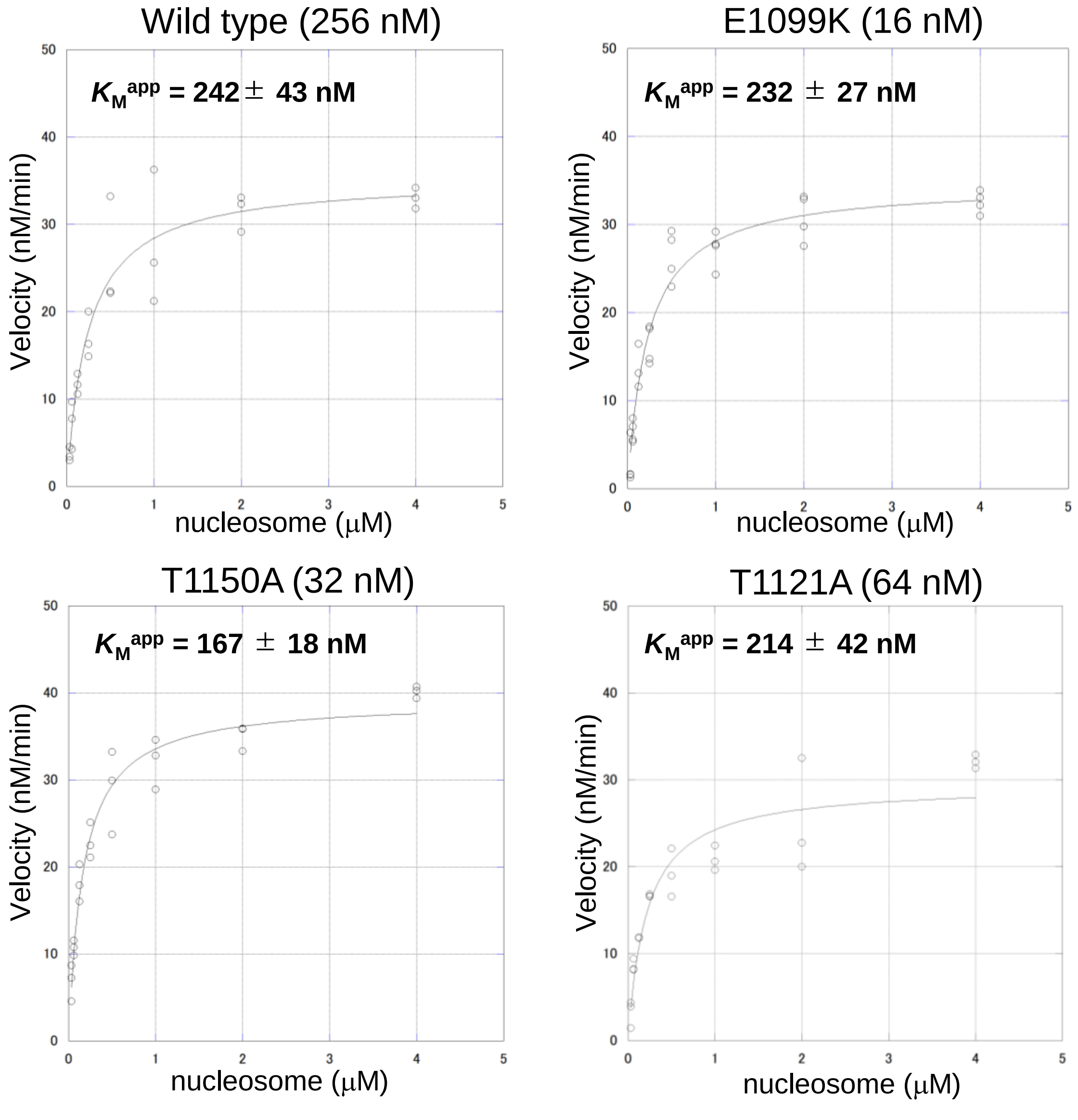

Supplementary Figure 8 Michaelis–Menten plots using the 185-bp nucleosome as a substrate. Source data are provided as a Source Data file.

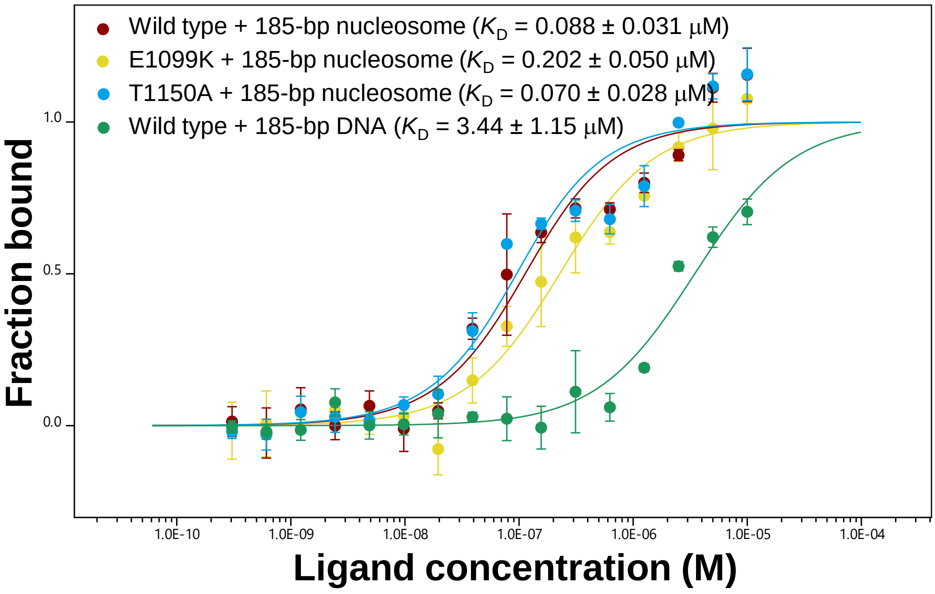

Supplementary Figure 9 Dose-response curves of the MST assay analyzing the interactions between wild-type or mutant NSD2 and the 185-bp nucleosome or naked 185-bp DNA. Data were presented as mean ± RMSE (root mean square error) of independently generated datasets (n = 3). The RMSE was calculated using MO.Affinity Analysis software (NanoTemper Technologies GmbH). Source data are provided as a Source Data file.

a

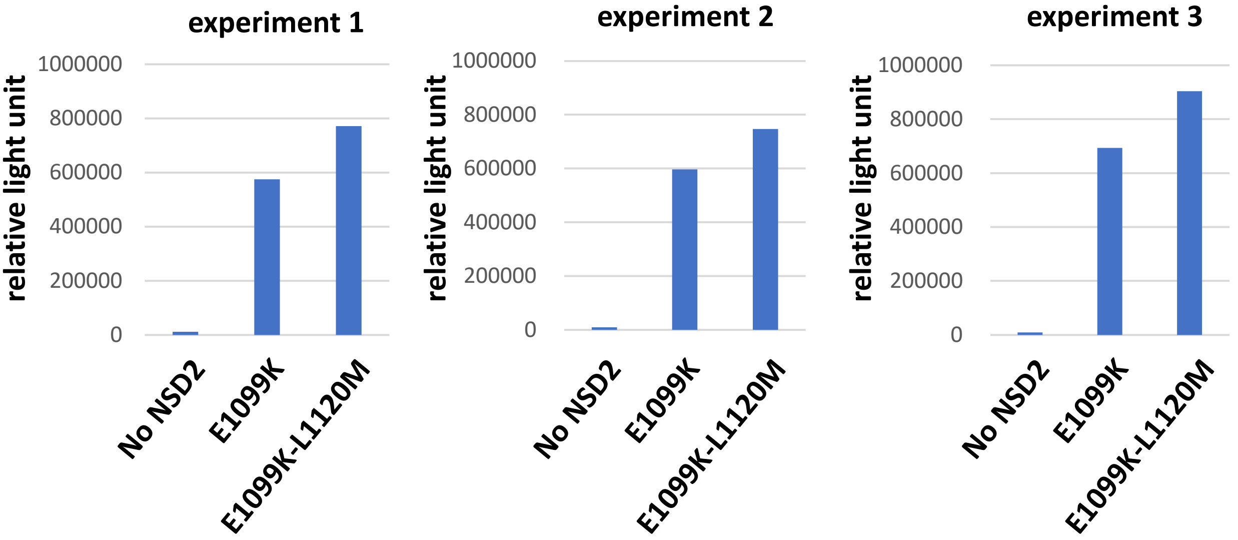

b

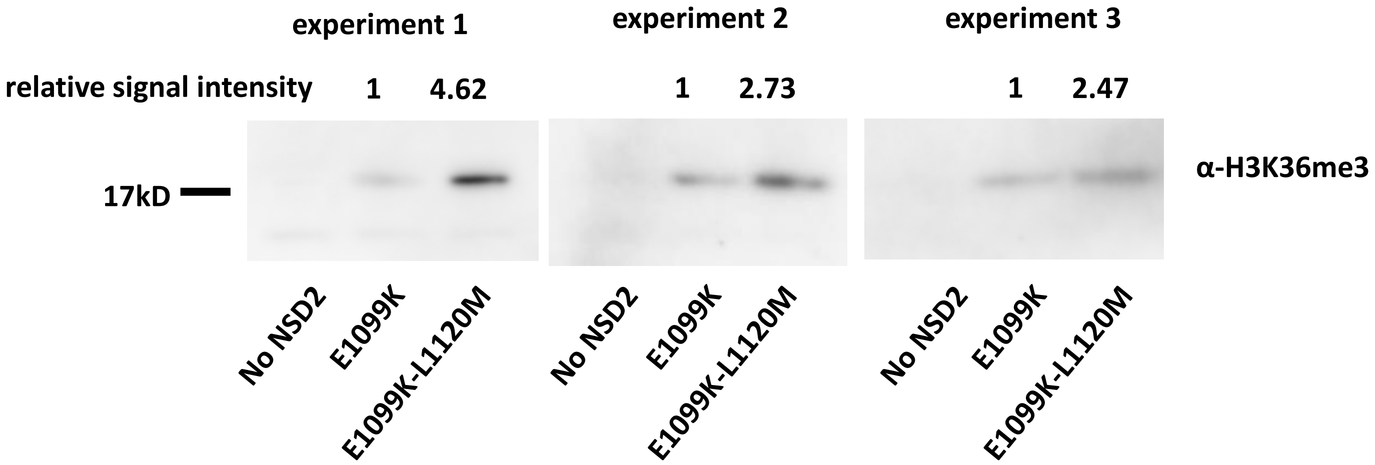

Supplementary Figure 10 Tri-methyltransferase assay. Experiments were repeated three times, and their results are shown. The Source data are provided as a Source Data file. **a**, Total methyltransferase activity of the E1099K single mutant and the E1099K-L1120M double mutant analyzed by the amount of the by-product SAH. **b**, Western blot analysis to detect H3K36me3 produced by the two mutants. The relative intensity of the H3K36me3 signal is shown.

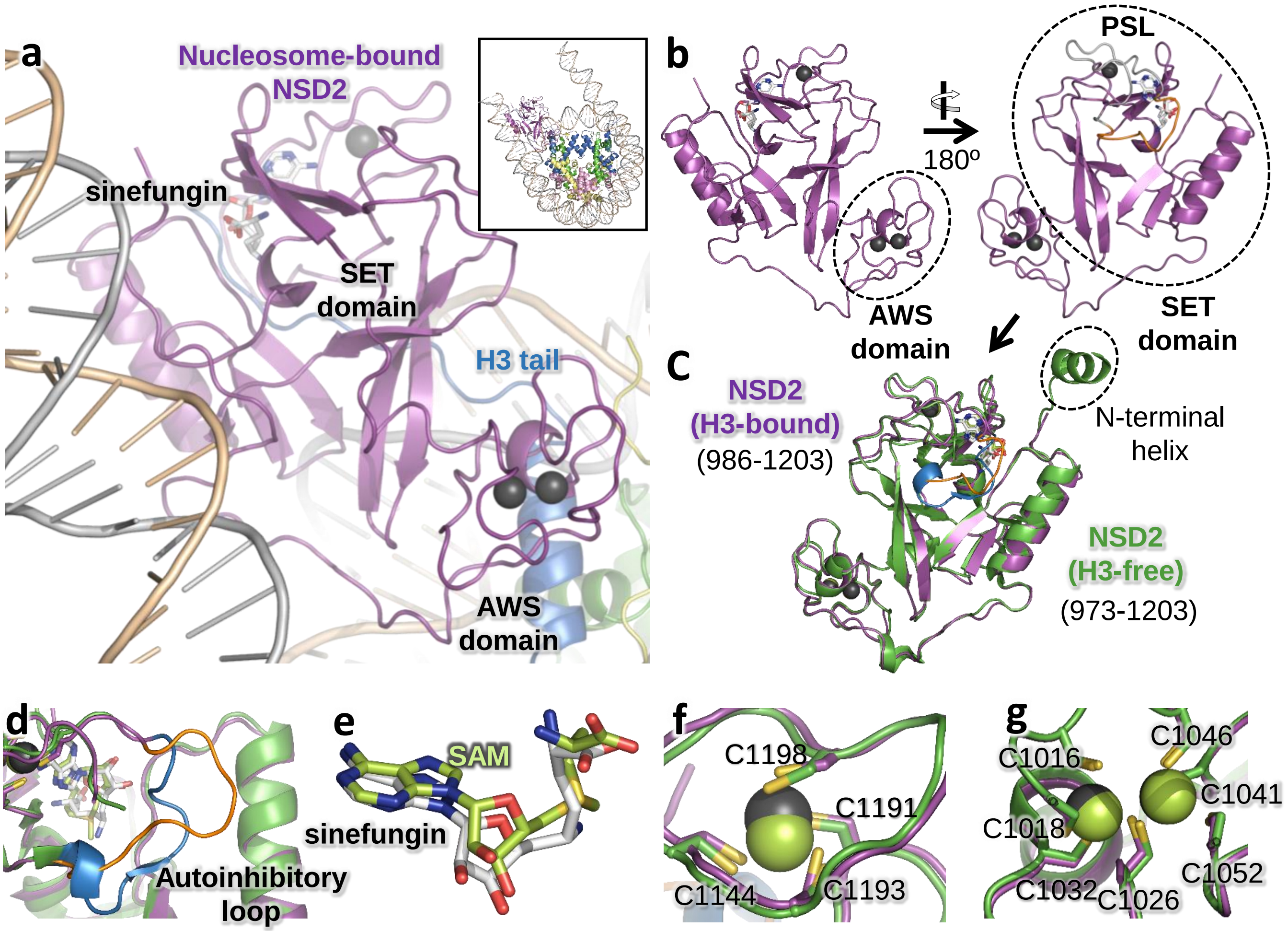

Supplementary Figure 11 Comparison of the nucleosome-bound NSD2 and H3-free NSD2 structures. a, Nucleosome-bound NSD2 structure shown in Fig. 1b. The structural elements are labeled. **b**, Two views of the NSD2 structure.The orientation of the left structure is kept as in a, while the right structure is rotated by 180°. AWS and SET domain structure are encircled in black and labeled. The post-SET loop (PSL) (gray) containing the autoinhibitory loop (orange) is shown on the right structure. **c**, Superimposition of the current NSD2 structure with that of the apo-NSD2 crystal structure (PDB 5LSU) shown in green. The structural orientation is the same as shown in right image of b. **d**, Comparison of the autoinhibitory loop conformations in the nucleosome-bound (orange) and H3-free NSD2 (blue) structures. **e**, SAM and sinefungin binding pose. **f**, Zn atom bound to the PSL. **g**, Zn atoms bound to the AWS domain of NSD2. In f and g, Zn atoms are colored gray and light green for the nucleosome-bound and apo structures, respectively.

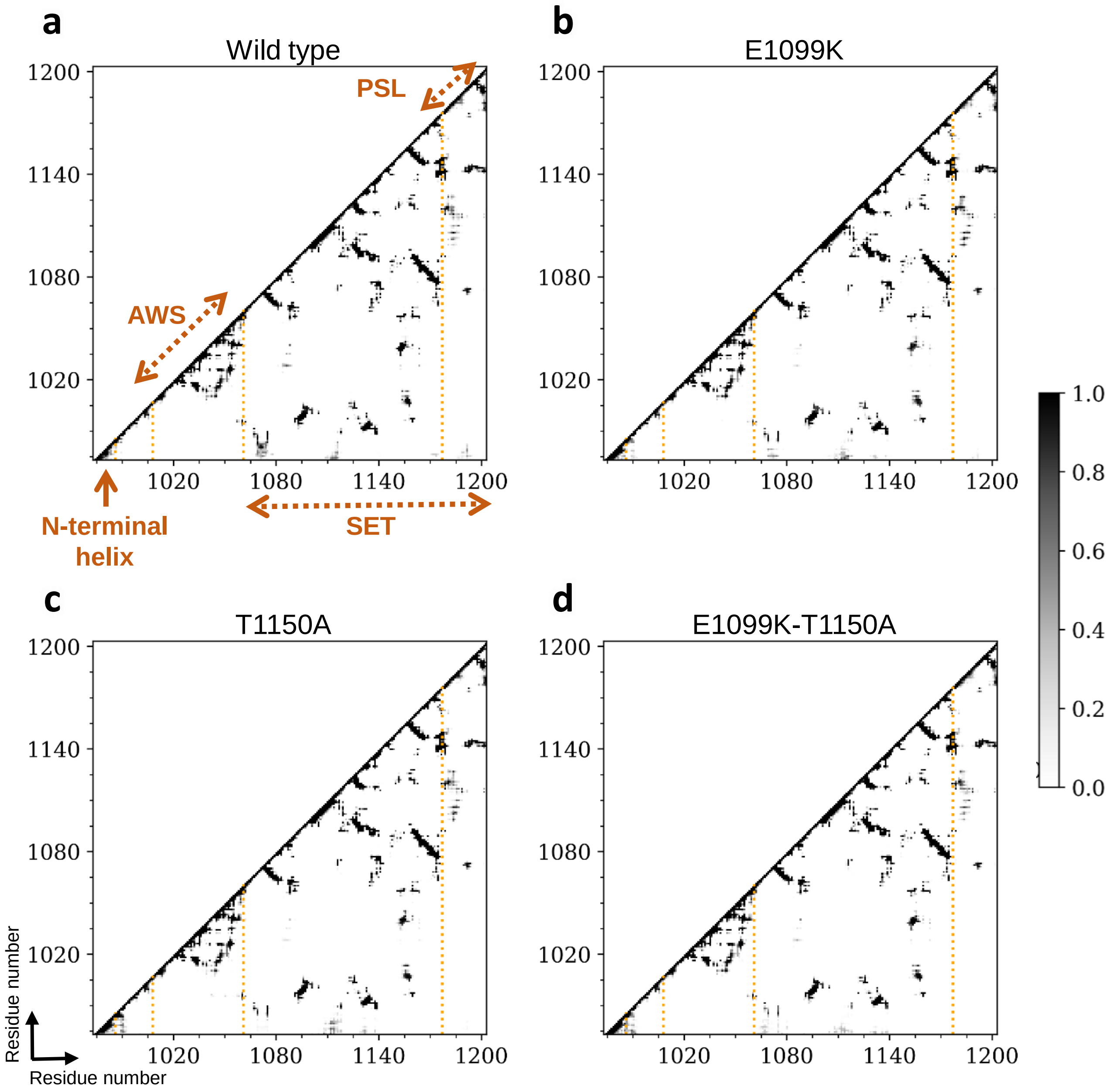

Supplementary Figure 12 Residue-wise contact map of the NSD2. a, wild-type **b**, E1099K **c**, T1150A, and **d**, E1099K-T1150A mutants. The color bar indicates the contact frequency (white: no contact and black: maximum contact) between the residues. A contact was counted only if the distance between any atom pair between two residues was less than or equal to 7 Å.

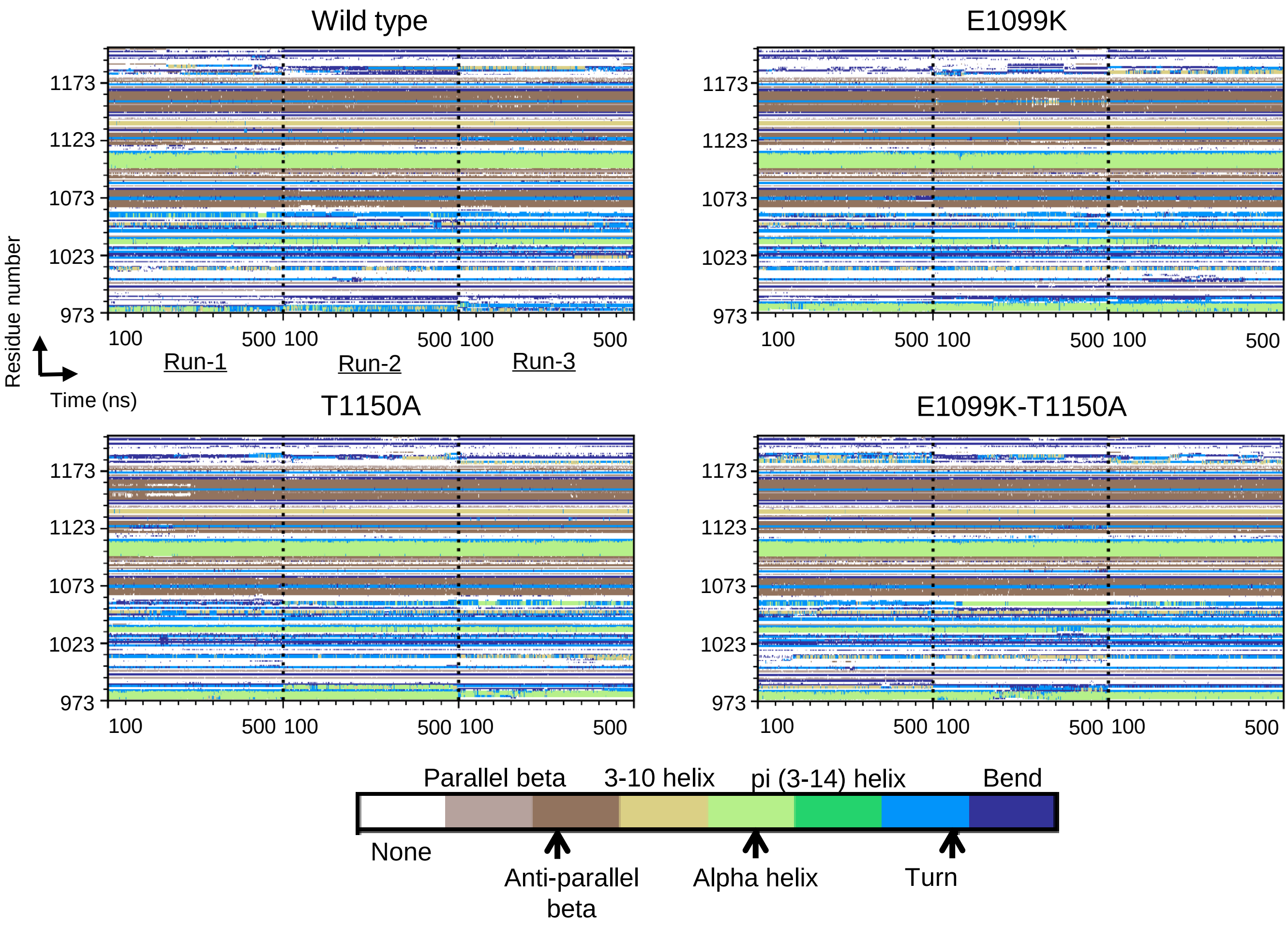

Supplementary Figure 13 Secondary structure time series for the last 400 ns of three independent runs of the NSD2 wild-type and mutants.

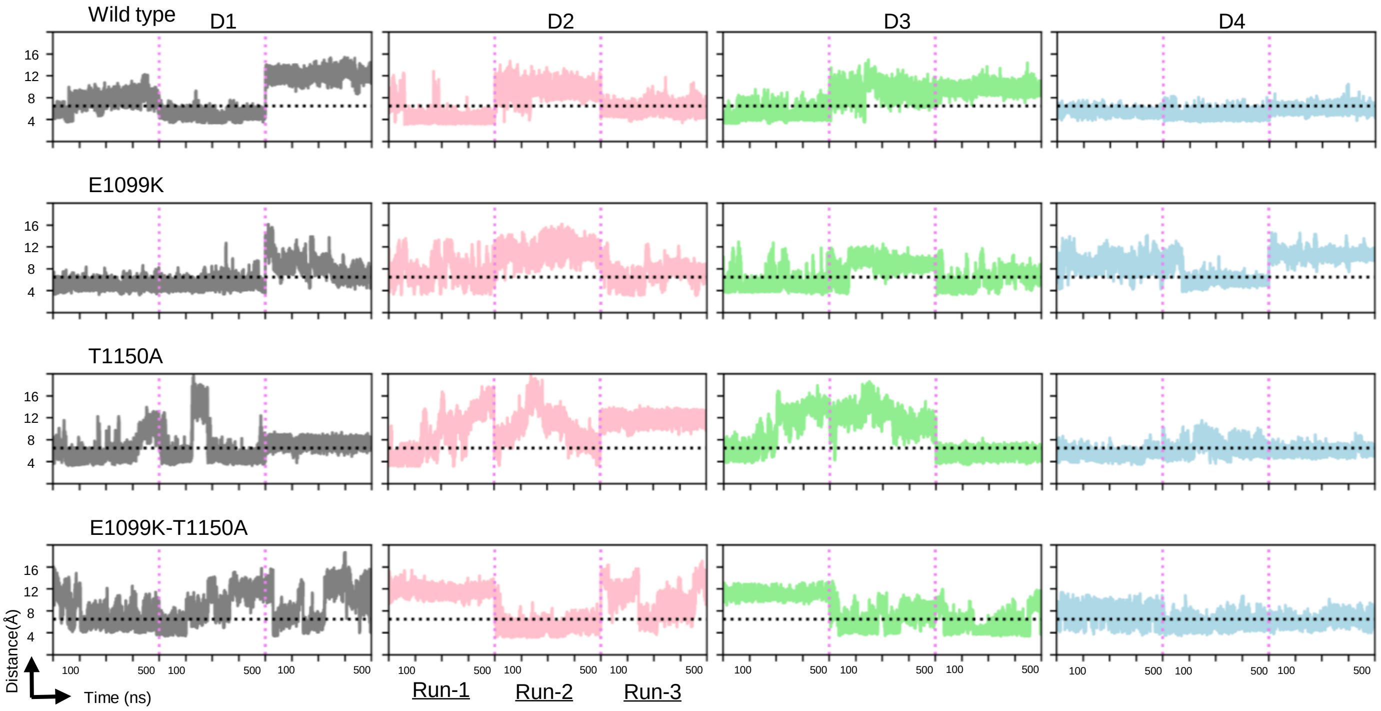

Supplementary Figure 14 Time course of distances D1, D2, D3, and D4 shown in Fig. 5d, used to define the open and closed conformations of the autoinhibitory loop for the wild-type and mutants. The black dotted line shows the cutoff criterion (6.5 Å) for being open or closed. Vertical pink dotted lines separate three independent runs.

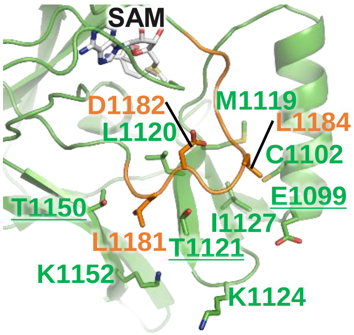

Supplementary Figure 15 Representative structure of wild-type NSD2 during MD simulation, in which the L1181 side chain forms hydrophobic interactions with T1121 and T1150 near the H3P38-binding patch.

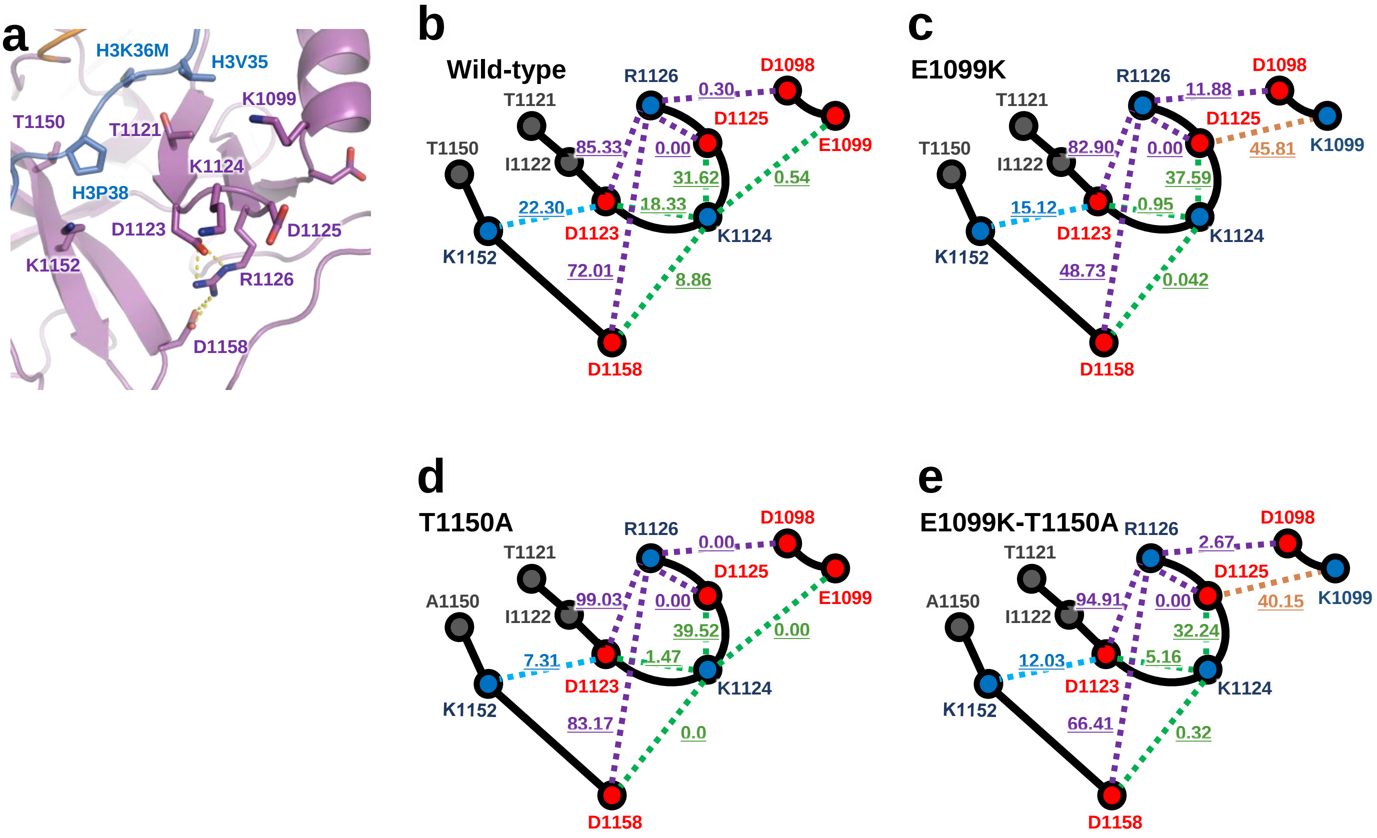

f

System	Wild-type		E1099K		T1150A		E1099K-T1150A	
D1123-K1152	Present	Absent	Present	Absent	Present	Absent	Present	Absent
Closed	22.14	76.99	13.97	76.30	7.03	86.71	8.54	72.67
Open	0.16	0.70	1.13	8.60	0.32	5.94	3.48	15.32

Supplementary Figure 16 Salt-bridge network around the autoinhibitory loop. a, Charged residues around the autoinhibitory loop of the apo NSD2 structure. **b-e**, Schematic representation of the salt-bridge network among the charged residues during the MD simulations of (b) the wild-type, (c) E1099K, (d) T1150A, and (e) E1099K-T1150A. The acidic and basic residues are shown in the red and blue spheres, respectively, and labeled. The dashed line with the labeled percentage shows the salt bridges between the residues. A salt bridge between a residue pair is defined as existing if the minimum distance between any side chain acidic-basic atom pair is less than or equal to 3.5 Å. **f**, Percent distribution of NSD2 structures in which the salt bridge between D1123 and K1152 is present or absent and the autoinhibitory loop presents a closed or open state.

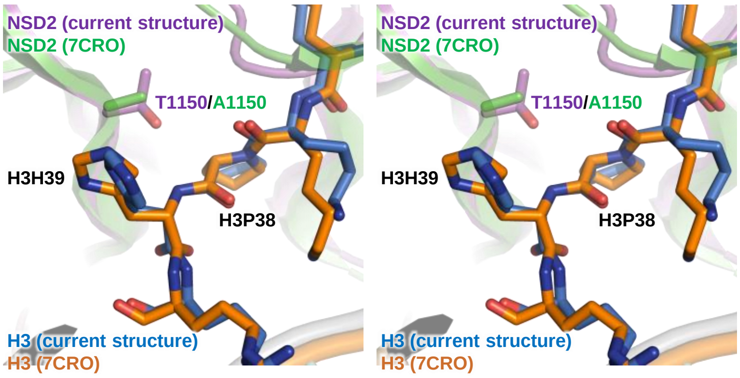

Supplementary Figure 17 Superposition of the current NSD2-nucleosome complex (bearing E1099K) and the NSD2 double mutant (E1099K-T1150A) bound to nucleosome (PDB ID 7CRO) shown in a stereo view.

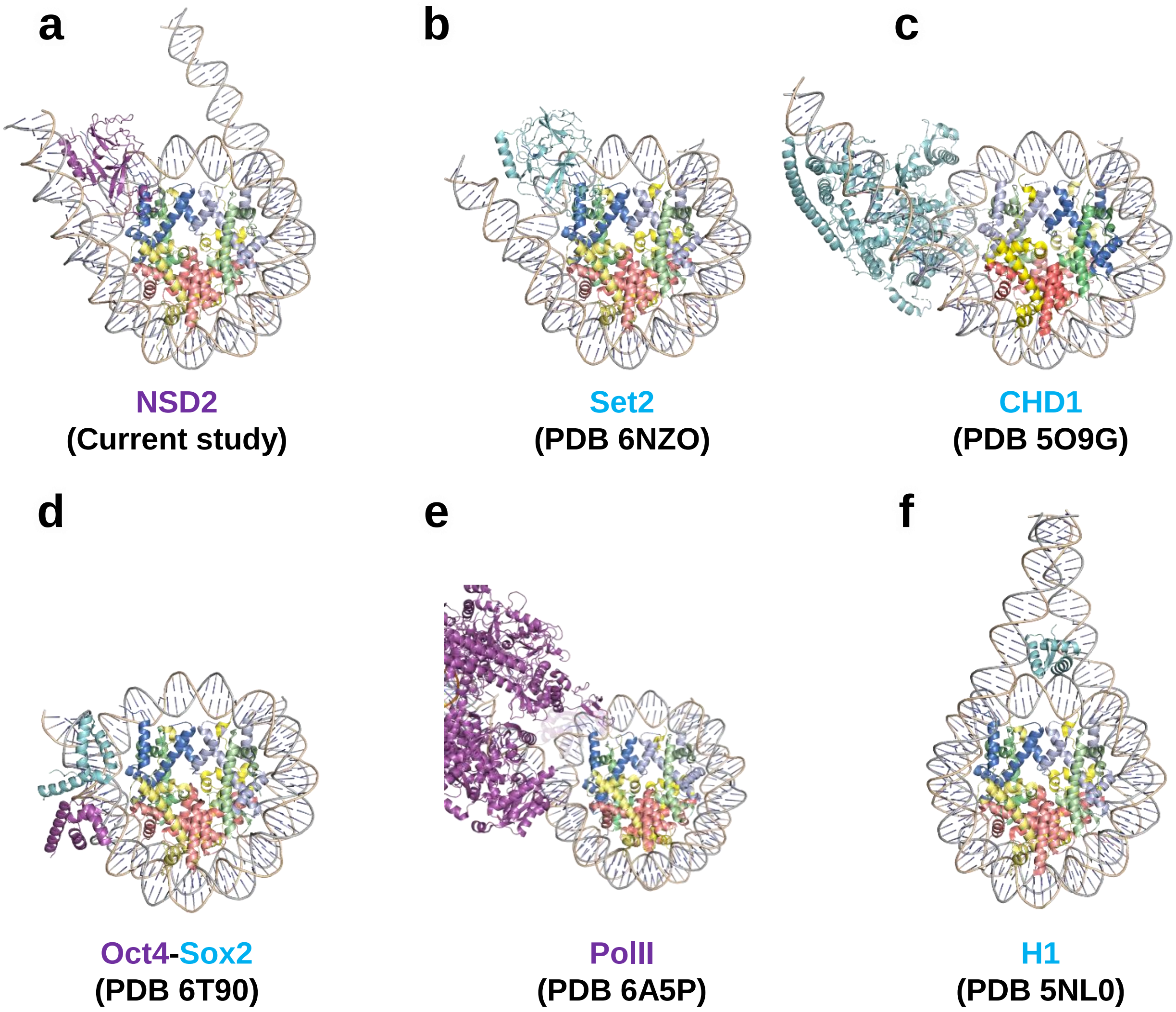

Supplementary Figure 18 Structures of chromatin factors bound to nucleosome. a, NSD2-nucleosome complex. **b**, Set2-nucleosome complex. **c**, CHD1-nucleosome complex. **d**, Oct4-Sox2 bound to a nucleosome. **e**, Yeast RNA polymerase II (PolII) bound to a nucleosome. **f,** Linker histone H1 bound to a nucleosome.

Supplementary Table 1 Cryo-EM data acquisition, refinement, and validation statistics

	25 mM KCl (1)	25 mM KCl (2)	50 mM KCl
**Data collection**			
Magnification	105,000	105,000	105,000
Voltage (kV)	300	300	300
Electron exposure ($e^-/Å^2$)	72.9	50	50
Frame	70	48	48
Defocus range (μm)	-0.8 to -1.6	-0.8 to -1.6	-0.8 to -1.6
Pixel size (Å)	0.83	0.83	0.83
No. of micrographs	2385	90	2008
**Data processing**	**NSD2-nucleosome**		
Symmetry imposed	C1		
Initial particle images	4,784,875		
Final particle images	136,108		
Map resolution (Å)	2.8		
FSC threshold	0.143		
**Refinement**			
Initial models (PDB IDs)	1KX5, 5LSU		
Model composition			
Non-hydrogen atoms	14,852		
Protein residues	983		
DNA residues	344		
Ligands	sinefungin: 1<br>zinc: 3		
B-factors ($Å^2$)			
Protein atoms	66.60		
DNA atoms	149.59		
Ligand atoms	113.03		
RMS deviations			
Bonds (Å)	0.010		
Angles (°)	0.702		
Validation			
MolProbity score	1.99		
Clashscore	7.59		
Ramachandran plot			
Favored (%)	94.82		
Allowed (%)	5.08		
Outliers (%)	0.10		
Model vs Data			
CC (mask)	0.87		
CC (box)	0.83		
CC (peaks)	0.78		
CC (volume)	0.86		
Mean CC for ligands	0.81		

Supplementary Table 2 List of MD simulation systems

No.	Referred name	Details of the system	Duration
**1**	Wild-type	NSD2 and SAM	500 ns × 3
**2**	E1099K	NSD2 E1099K and SAM	500 ns × 3
**3**	T1150A	NSD2 T1150A and SAM	500 ns × 3
**4**	E1099K-T1150A	NSD2 with two mutations E1099K and T1150, and SAM	500 ns × 3

NSD2 refers to residues 973 to 1203, constituting N-terminal helix, AWS domain, SET domain, and PS loop. SAM: S-adenosyl methionine. The starting structure of NSD2 was taken from the cryo-EM structure reported in the current study.

Supplementary Table 3 Open-closed dynamics of the autoinhibitory loop

**MD system**	**% of individual locks released**				**All released (%)**
	**L1184@Cγ-C1102@Sγ (D1)**	**C1183@Sγ-C1102@Sγ (D2)**	**C1183@Sγ-T1121@Cβ (D3)**	**L1181@Cγ-T1121@Cβ (D4)**	
**Wild-type**	**60.4**	**44.1**	**68.5**	**5.1**	**0.86**
**E1099K**	**30.3**	**81.2**	**50.5**	**74.0**	**9.7**
**T1150A**	**50.4**	**85.1**	**58.3**	**21.1**	**6.3**
**E1099K-T1150A**	**67.1**	**67.5**	**61.0**	**43.8**	**18.8**

Note: A lock is operational when the two atoms indicated are within 6.5 Å distance cutoff; otherwise it is released. When all the locks are released, the autoinhibitory loop is considered "open".

Supplementary Table 4 Percent distribution of NSD2 structures among the sixteen possible states

States	D1	D2	D3	D4	Wild-type (%)	E1099K (%)	T1150A (%)	E1099K-T1150A (%)
State 1	1	1	1	1	1.69	0.40	6.88	6.25
State 2	0	1	1	1	23.92	0.030	1.93	10.84
State 3	1	0	1	1	4.98	0.21	2.17	3.36
State 4	1	1	0	1	0.35	0.07	3.42	2.11
State 5	1	1	1	0	0.018	7.63	0.25	1.03
State 6	0	0	1	1	0.25	0.008	24.62	8.44
State 7	0	1	0	1	26.23	0.009	2.13	10.77
State 8	0	1	1	0	0.39	9.83	0.09	0.52
State 9	1	0	0	1	32.10	23.88	27.59	4.66
State 10	1	0	1	0	0.24	22.81	0.72	1.47
State 11	1	1	0	0	0.004	0.18	0.07	0.08
State 12	0	0	0	1	5.42	1.39	10.16	9.74
State 13	0	1	0	0	3.25	0.65	0.14	0.96
State 14	0	0	1	0	0.036	8.61	5.08	7.05
State 15	1	0	0	0	0.26	14.57	8.50	13.92
State 16	0	0	0	0	0.86	9.73	6.25	18.80
Entropy ($-\sum_{i=1}^{16} p_i \log p_i$)					1.68	1.97	2.08	2.39

1 = Operational, 0 = released; State 16 = All the locks released and the autoinhibitory loop is open.
For entropy, p_i represents the probability of the *i*-th state.

Supplementary Table 5 Primers used in this study

NSD2-E1099KF	GATCGACAAGGAGGAGTGCATGGCGA
NSD2-E1099KR	TCCTCCTTGTCGATCAGCTCCCCAAC
HsH3K36MF2	GGGGTCATGAAGCCTCATCGCTATCGT
HsH3K36MR2	GAGGCTTCATGACCCCACCCGTGGCCG
282F	CGGGATCCTAATGACCAAGGAAAGC
282R	GGGAGCTCGGAACACTATCCGAC
185F	GACCCTATACGCGGCCGCCCTGG
185R	GTCGCTGTTCAATACATGCACAGGATG
NSD2K992AF	CCATACGCGCACATCAAGGTGAA
NSD2K992AR	GATGTGCGCGTATGGTGGGGGCTT
NSD2K995-998AF	TCGCGGTGAATGCGCCTTACGGGAAAGTCCA
NSD2K995-998AR	GCGCATTCACCGCGATGTGCGCGTATGGTGG
NSD2N1034AF	TGTCTGGCCAGGATGCTGATGTT
NSD2N1034AR	CATCCTGGCCAGACACTCCGAATC
NSD2E1099RF	ATCGACCGGGAGGAGTGCATGGCGAG
NSD2E1099RR	CTCCTCCCGGTCGATCAGCTCCCCAAC
NSD2E1099QF	GATCGACCAGGAGGAGTGCATGGCGA
NSD2E1099QR	TCCTCCTGGTCGATCAGCTCCCCAAC
NSD2E1099AF	ATCGACGCGGAGGAGTGCATGGCGAG
NSD2E1099AR	CTCCTCCGCGTCGATCAGCTCCCCAA
NSD2T1121AF	CATGCTCGCTATAGACAAGGACCGTA
NSD2T1121AR	TCTATAGCGAGCATGTAGAAGTGGGT
NSD2T1150AF	CTGTGAGGCCCTCAAGTGGACAGTGA
NSD2T1150AR	TTGAGGGCCTCACAGTTGGGCTGGCA
NSD2K1152AF	ACCCTCGCGTGGACAGTGAATGG
NSD2K1152AR	TGTCCACGCGAGGGTCTCACAGTT
NSD2T1154AF	AAGTGGGCAGTGAATGGGGACAC
NSD2T1154AR	TTCACTGCCCACTTGAGGGTCTC
NSD2D1182AF	AACCTCGCTTGTCTGGGCAATGAAAA
NSD2D1182AR	CAGACAAGCGAGGTTGTAGTTAAAAG
NSD2D1182NF	CAACCTCAATTGTCTGGGCAATGAAA
NSD2D1182NR	AGACAATTGAGGTTGTAGTTAAAAGT

Supplementary Table 6 Sequences of protein-coding regions used in this study

His6-SUMOstar-H2A	ATGAATCACAAAGTGCATCATCACCACCATCATGGGTCCCT GCAGGACTCAGAAGTCAATCAAGAAGCTAAGCCAGAGGTC AAGCCAGAAGTCAAGCCTGAGACTCACATCAATTTAAAGGT GTCCGATGGATCTTCAGAGATCTTCTTCAAGATCAAAAAGA CCACTCCTTTAAGAAGGCTGATGGAAGCGTTCGCTAAAAG ACAGGGTAAGGAAATGGACTCCTTAACGTTCTTGTACGACG GTATTGAAATTCAAGCTGATCAGACCCCTGAAGATTTGGAC ATGGAGGATAACGATATTATTGAGGCTCACAGAGAACAGAT TGGAGGTAGCGGTCGTGGAAAGGGGGGTAAAGGATTAGG GAAAGGAGGAGCAAAACGCCACCGCAAAGTATTACGTGAC AATATCCAAGGTATCACTAAGCCTGCGATCCGTCGCTTGGC TCGTCGCGGAGGCGTTAAACGCATCTCGGGTCTTATTTATG AAGAAACCCGTGGAGTTCTTAAGGTATTCCTTGAAAACGTC ATCCGTGACGCTGTTACTTACACCGAGCACGCTAAACGTAA AACGGTCACGGCAATGGATGTTGTATATGCACTGAAACGTC AGGGGCGTACTTTGTATGGATTCGGGGGTTAA
His6-H2B	ATGGGCAGCAGCCATCATCATCATCATCACAGCAGCGGCC TGGTGCCGCGCGGCAGCCATATGCCAGAGCCAGCGAAGT CTGCTCCCGCCCCGAAAAAGGGCTCCAAGAAGGCGGTGA CTAAGGCGCAGAAGAAAGACGGCAAGAAGCGCAAGCGCA GCCGCAAGGAGAGCTATTCCATCTATGTGTACAAGGTTCTG AAGCAGGTCCACCCTGACACCGGCATTTCGTCCAAGGCCA TGGGCATCATGAATTCGTTTGTGAACGACATTTTCGAGCGC ATCGCAGGTGAGGCTTCCCGCCTGGCGCATTACAACAAGC GCTCGACCATCACCTCCAGGGAGATCCAGACGGCCGTGC GCCTGCTGCTGCCTGGGGAGTTGGCCAAGCACGCCGTGT CCGAGGGTACTAAGGCCGTCACCAAGTACACCAGCGCTAA GTAG
His6-SUMOstar-H4/H3	ATGAATCACAAAGTGCATCATCACCACCATCATGGGTCCCT GCAGGACTCAGAAGTCAATCAAGAAGCTAAGCCAGAGGTC AAGCCAGAAGTCAAGCCTGAGACTCACATCAATTTAAAGGT GTCCGATGGATCTTCAGAGATCTTCTTCAAGATCAAAAAGA CCACTCCTTTAAGAAGGCTGATGGAAGCGTTCGCTAAAAG ACAGGGTAAGGAAATGGACTCCTTAACGTTCTTGTACGACG GTATTGAAATTCAAGCTGATCAGACCCCTGAAGATTTGGAC

	ATGGAGGATAACGATATTATTGAGGCTCACAGAGAACAGAT TGGAGGTAGCGGTCGTGGAAAGGGAGGTAAAGGATTAGG GAAAGGAGGAGCAAAACGCCACCGCAAAGTATTACGTGAC AATATCCAAGGTATCACTAAGCCTGCGATCCGTCGCTTGGC TCGTCGCGGAGGCGTTAAACGCATCTCGGGTCTTATTTATG AAGAAACCCGTGGAGTTCTTAAGGTATTCCTTGAAAACGTC ATCCGTGACGCTGTTACTTACACCGAGCACGCTAAACGTAA AACGGTCACGGCAATGGATGTTGTATATGCACTGAAACGTC AGGGGCGTACTTTGTATGGATTCGGGGGTTAAGCGGCCGC ATAATGCTTAAGTCGAACAGAAAGTAATCGTATTGTACACGG CCGCATAATCGAAATTAATACGACTCACTATAGGGGAATTGT GAGCGGATAACAATTCCCCATCTTAGTATATTAGTTAAGTATA AGAAGGAGATATACATATGGCACGCACAAAACAGACGGCC CGCAAGTCTACCGGCGGCAAGGCTCCACGCAAACAACTT GCGACCAAAGCGGCGCGTAAGTCGGCTCCGGCCACGGGT GGGGTCAAAAAGCCTCATCGCTATCGTCCGGGAACTGTCG CGCTTCGTGAAATTCGTCGCTACCAGAAATCCACTGAATTG CTTATTCGTAAGTTACCCTTCCAGCGTTTGGTCCGTGAGAT TGCTCAGGACTTCAAAACGGACTTGCGCTTCCAGAGTAGC GCCGTCATGGCCCTGCAGGAGGCCTGTGAAGCATATCTTG TAGGGCTTTTTGAAGATACCAACCTGTGTGCCATTCATGCT AAACGCGTAACTATCATGCCCAAAGACATCCAGTTGGCTCG TCGTATTCGCGGTGAACGTGCCTAA
His6-SUMOstar-NSD2E1099K-H4/H3K36M	ATGAATCACAAAGTGCATCATCACCACCATCATGGGTCCCT GCAGGACTCAGAAGTCAATCAAGAAGCTAAGCCAGAGGTC AAGCCAGAAGTCAAGCCTGAGACTCACATCAATTTAAAGGT GTCCGATGGATCTTCAGAGATCTTCTTCAAGATCAAAAAGA CCACTCCTTTAAGAAGGCTGATGGAAGCGTTCGCTAAAAG ACAGGGTAAGGAAATGGACTCCTTAACGTTCTTGTACGACG GTATTGAAATTCAAGCTGATCAGACCCCTGAAGATTTGGAC ATGGAGGATAACGATATTATTGAGGCTCACAGAGAACAGAT TGGAGGTAAGCTTCAGAGGGAAGCCCGAGAAACACAGGA GAGCGAGCGCAAGCCCCCACCATACAAGCACATCAAGGTG AATAAGCCTTACGGGAAAGTCCAGATCTACACAGCGGATAT TTCAGAAATCCCTAAGTGCAACTGCAAGCCCACAGATGAG AATCCTTGTGGCTTTGATTCGGAGTGTCTGAACAGGATGCT

	GATGTTTGAGTGCCACCCGCAGGTGTGTCCCGCGGGCGA<br>GTTCTGCCAGAACCAGTGCTTCACCAAGCGCCAGTACCCA<br>GAGACCAAGATCATCAAGACAGATGGCAAAGGGTGGGGC<br>CTGGTCGCCAAGAGGGACATCAGAAAGGGAGAATTTGTTA<br>ACGAGTACGTTGGGGAGCTGATCGACAAGGAGGAGTGCAT<br>GGCGAGAATCAAGCACGCACACGAGAACGACATCACCCAC<br>TTCTACATGCTCACTATAGACAAGGACCGTATAATAGACGCT<br>GGCCCCAAAGGAAACTACTCTCGATTTATGAATCACAGCTG<br>CCAGCCCAACTGTGAGACCCTCAAGTGGACAGTGAATGG<br>GGACACTCGTGTGGGCCTGTTTGCCGTCTGTGACATTCCT<br>GCAGGGACGGAGCTGACTTTTAACTACAACCTCGATTGTCT<br>GGGCAATGAAAAAACGGTCTGCCGGTGTGGAGCCTCCAAT<br>TGCAGTGGATTCCTCGGGGATAGACCAAAGACCTCGACGA<br>CCCTTTCATCAGAGGAAAAGGGCAAAAAGACCAAGAAGAA<br>AACGAGGGGTGGTGGATCTGGAGGTGGTAGTGGTGGAGG<br>ATCAGGAGGAGGTAGTGGTGGTGGTAGTGGAGGTGGATCT<br>GGTGGAGGTAGTGGAGGAGGATCTAGCGGTCGTGGAAAG<br>GGAGGTAAAGGATTAGGGAAAGGAGGAGCAAAACGCCAC<br>CGCAAAGTATTACGTGACAATATCCAAGGTATCACTAAGCCT<br>GCGATCCGTCGCTTGGCTCGTCGCGGAGGCGTTAAACGC<br>ATCTCGGGTCTTATTTATGAAGAAACCCGTGGAGTTCTTAA<br>GGTATTCCTTGAAAACGTCATCCGTGACGCTGTTACTTACA<br>CCGAGCACGCTAAACGTAAAACGGTCACGGCAATGGATGT<br>TGTATATGCACTGAAACGTCAGGGGCGTACTTTGTATGGAT<br>TCGGGGGTTAAGCGGCCGCATAATGCTTAAGTCGAACAGA<br>AAGTAATCGTATTGTACACGGCCGCATAATCGAAATTAATAC<br>GACTCACTATAGGGGAATTGTGAGCGGATAACAATTCCCCA<br>TCTTAGTATATTAGTTAAGTATAAGAAGGAGATATACATATGG<br>CACGCACAAAACAGACGGCCCGCAAGTCTACCGGCGGCA<br>AGGCTCCACGCAAACAACTTGCGACCAAAGCGGCGCGTA<br>AGTCGGCTCCGGCCACGGGTGGGGTCATGAAGCCTCATC<br>GCTATCGTCCGGGAACTGTCGCGCTTCGTGAAATTCGTCG<br>CTACCAGAAATCCACTGAATTGCTTATTCGTAAGTTACCCTT<br>CCAGCGTTTGGTCCGTGAGATTGCTCAGGACTTCAAAACG<br>GACTTGCGCTTCCAGAGTAGCGCCGTCATGGCCCTGCAG<br>GAGGCCTGTGAAGCATATCTTGTAGGGCTTTTTGAAGATAC

	CAACCTGTGTGCCATTCATGCTAAACGCGTAACTATCATGC CCAAAGACATCCAGTTGGCTCGTCGTATTCGCGGTGAACG TGCCTAA
Twin-Strep-His6-NSD2	ATGAATCACAAAGTGTGGAGCCATCCGCAGTTTGAAAAAG GAGGTGGCTCAGGTGGAGGATCTGGTGGTAGTGCTTGGA GCCATCCGCAGTTTGAAAAAGGAAGTCATCACCATCACCA CCATGGATCACTCGAGGTTCTGTTCCAGGGGCCCAAGCTT CAGAGGGAAGCCCGAGAAACACAGGAGAGCGAGCGCAAG CCCCCACCATACAAGCACATCAAGGTGAATAAGCCTTACGG GAAAGTCCAGATCTACACAGCGGATATTTCAGAAATCCCTA AGTGCAACTGCAAGCCCACAGATGAGAATCCTTGTGGCTT TGATTCGGAGTGTCTGAACAGGATGCTGATGTTTGAGTGC CACCCGCAGGTGTGTCCCGCGGGCGAGTTCTGCCAGAAC CAGTGCTTCACCAAGCGCCAGTACCCAGAGACCAAGATCA TCAAGACAGATGGCAAAGGGTGGGGCCTGGTCGCCAAGA GGGACATCAGAAAGGGAGAATTTGTTAACGAGTACGTTGG GGAGCTGATCGACGAGGAGGAGTGCATGGCGAGAATCAA GCACGCACACGAGAACGACATCACCCACTTCTACATGCTC ACTATAGACAAGGACCGTATAATAGACGCTGGCCCCAAAGG AAACTACTCTCGATTTATGAATCACAGCTGCCAGCCCAACT GTGAGACCCTCAAGTGGACAGTGAATGGGGACACTCGTGT GGGCCTGTTTGCCGTCTGTGACATTCCTGCAGGGACGGA GCTGACTTTTAACTACAACCTCGATTGTCTGGGCAATGAAA AAACGGTCTGCCGGTGTGGAGCCTCCAATTGCAGTGGATT CCTCGGGGATAGACCAAAGACCTCGACGACCCTTTCATCA GAGGAAAAGGGCAAAAAGACCAAGAAGAAAACGAGGTGA
