# Supplementary material for: Structural basis of the regulation of the normal and oncogenic methylation of nucleosomal histone H3 Lys36 by NSD2
Source: Nat Commun. 2021 Nov 15;12:6605. doi: 10.1038/s41467-021-26913-5 (PMC8593083; doi:10.1038/s41467-021-26913-5)
Supplement: Supplementary file 2 — Description of Additional Supplementary Files [file 41467_2021_26913_MOESM2_ESM.pdf]

Description of Additional Supplementary Files

Supplementary Movie 1: Representative movie of the E1099K mutant showing autoinhibitory loop movement as it changes from a closed state to an open state, observed during the MD simulation. The carbon atoms of T1150 and E1099K are colored cyan. The backbone of the autoinhibitory loop is colored orange. The carbon atoms of the residues in the H3V35-binding patch are colored yellow. L1181, C1183, and L1184 are colored according to the state of the autoinhibitory loop (red, all locks operational; white, locks partially released; blue, all locks released).
