# Supplementary material for: Structural basis of the regulation of the normal and oncogenic methylation of nucleosomal histone H3 Lys36 by NSD2
Source: Nat Commun. 2021 Nov 15;12:6605. doi: 10.1038/s41467-021-26913-5 (PMC8593083; doi:10.1038/s41467-021-26913-5)
Supplement: Supplementary file 4 — Reporting summary [file 41467_2021_26913_MOESM4_ESM.pdf]

nature portfolio

Corresponding author(s): Toru Sengoku

Last updated by author(s): Oct 21, 2021

Reporting Summary

Nature Portfolio wishes to improve the reproducibility of the work that we publish. This form provides structure for consistency and transparency in reporting. For further information on Nature Portfolio policies, see our Editorial Policies and the Editorial Policy Checklist.

Statistics

For all statistical analyses, confirm that the following items are present in the figure legend, table legend, main text, or Methods section.

n/a	Confirmed	
☐	☒	The exact sample size (n) for each experimental group/condition, given as a discrete number and unit of measurement
☐	☒	A statement on whether measurements were taken from distinct samples or whether the same sample was measured repeatedly
☒	☐	The statistical test(s) used AND whether they are one- or two-sided<br>*Only common tests should be described solely by name; describe more complex techniques in the Methods section.*
☒	☐	A description of all covariates tested
☒	☐	A description of any assumptions or corrections, such as tests of normality and adjustment for multiple comparisons
☐	☒	A full description of the statistical parameters including central tendency (e.g. means) or other basic estimates (e.g. regression coefficient) AND variation (e.g. standard deviation) or associated estimates of uncertainty (e.g. confidence intervals)
☒	☐	For null hypothesis testing, the test statistic (e.g. F, t, r) with confidence intervals, effect sizes, degrees of freedom and P value noted<br>*Give P values as exact values whenever suitable.*
☒	☐	For Bayesian analysis, information on the choice of priors and Markov chain Monte Carlo settings
☒	☐	For hierarchical and complex designs, identification of the appropriate level for tests and full reporting of outcomes
☒	☐	Estimates of effect sizes (e.g. Cohen's d, Pearson's r), indicating how they were calculated

Our web collection on statistics for biologists contains articles on many of the points above.

Software and code

Policy information about availability of computer code

Data collection	SerialEM Version 3.8.0 beta
Data analysis	RELION 3.1; MotionCor2 v1.3.1; Gctf v1.18; cryoSPARC v2.15.0; Chimera 1.14; ChimeraX 1.0; Coot 0.8.9.2; Phenix 1.18.2; PyMOL 2.5.0a0; KaleidaGraph 4.5.3; MO. Affinity Analysis ver 2.3, Amber18, Gaussian 16, SHAKE

For manuscripts utilizing custom algorithms or software that are central to the research but not yet described in published literature, software must be made available to editors and reviewers. We strongly encourage code deposition in a community repository (e.g. GitHub). See the Nature Portfolio guidelines for submitting code & software for further information.

Data

Policy information about availability of data

All manuscripts must include a data availability statement. This statement should provide the following information, where applicable:

- Accession codes, unique identifiers, or web links for publicly available datasets
- A description of any restrictions on data availability
- For clinical datasets or third party data, please ensure that the statement adheres to our policy

The density map and model coordinates have been deposited to Electron Microscopy Data Bank (accession number EMD-31015) and Protein Data Bank (accession number 7E8D), respectively. Source data are provided with this paper.

Field-specific reporting

Please select the one below that is the best fit for your research. If you are not sure, read the appropriate sections before making your selection.

- [x] Life sciences
- [ ] Behavioural & social sciences
- [ ] Ecological, evolutionary & environmental sciences

For a reference copy of the document with all sections, see nature.com/documents/nr-reporting-summary-flat.pdf

Life sciences study design

All studies must disclose on these points even when the disclosure is negative.

Sample size	136,108 particles were finally selected by using programs cryoSPARC (Ab-initio reconstruction and Hetero refinement modules) and Relion (Focused 3D classification module) to reconstitute the 2.8 A resolution map, which was sufficient to build an atomic model of the NSD2-nucleosome complex. MTase assays and MST assays were repeated independently at least three times.
Data exclusions	During the cryo-EM data analysis, bad particles were excluded with the programs cryoSPARC and Relion. For biochemical analyses, no data were excluded.
Replication	Biochemical experiments were repeated at least three times. All attempts at replication were successful.
Randomization	Samples were not allocated to groups.
Blinding	Investigators were not blinded during data acquisition and analysis because the results were quantitative and did not require subjective interpretation.

Reporting for specific materials, systems and methods

We require information from authors about some types of materials, experimental systems and methods used in many studies. Here, indicate whether each material, system or method listed is relevant to your study. If you are not sure if a list item applies to your research, read the appropriate section before selecting a response.

Materials & experimental systems

n/a	Involved in the study	
[ ]	[x]	Antibodies
[x]	[ ]	Eukaryotic cell lines
[x]	[ ]	Palaeontology and archaeology
[x]	[ ]	Animals and other organisms
[x]	[ ]	Human research participants
[x]	[ ]	Clinical data
[x]	[ ]	Dual use research of concern

Methods

n/a	Involved in the study	
[x]	[ ]	ChIP-seq
[x]	[ ]	Flow cytometry
[x]	[ ]	MRI-based neuroimaging

Antibodies

Antibodies used	anti tri-methyl-histone H3 (Lys36) (D5A7) XP Rabbit mAb #4909, Cell Signaling Technology
Validation	The antibody was validated in western blot analysis using extracts from HeLa, C2C12, C6, and COS cells by the manufacturer (https://www.cellsignal.jp/products/primary-antibodies/tri-methyl-histone-h3-lys36-d5a7-xp-rabbit-mab/4909).
